# Functionathon: a manual data mining workflow to generate functional hypotheses for uncharacterized human proteins and its application by undergraduate students

**DOI:** 10.1093/database/baab046

**Published:** 2021-07-28

**Authors:** Paula Duek, Camille Mary, Monique Zahn-Zabal, Amos Bairoch, Lydie Lane

**Affiliations:** CALIPHO group, SIB Swiss Institute of Bioinformatics; Department of microbiology and molecular medicine, Faculty of medicine, University of Geneva, Geneva, Switzerland; Department of microbiology and molecular medicine, Faculty of medicine, University of Geneva, Geneva, Switzerland; CALIPHO group, SIB Swiss Institute of Bioinformatics; CALIPHO group, SIB Swiss Institute of Bioinformatics; Department of microbiology and molecular medicine, Faculty of medicine, University of Geneva, Geneva, Switzerland; CALIPHO group, SIB Swiss Institute of Bioinformatics; Department of microbiology and molecular medicine, Faculty of medicine, University of Geneva, Geneva, Switzerland

## Abstract

About 10% of human proteins have no annotated function in protein knowledge bases. A workflow to generate hypotheses for the function of these uncharacterized proteins has been developed, based on predicted and experimental information on protein properties, interactions, tissular expression, subcellular localization, conservation in other organisms, as well as phenotypic data in mutant model organisms. This workflow has been applied to seven uncharacterized human proteins (C6orf118, C7orf25, CXorf58, RSRP1, SMLR1, TMEM53 and TMEM232) in the frame of a course-based undergraduate research experience named Functionathon organized at the University of Geneva to teach undergraduate students how to use biological databases and bioinformatics tools and interpret the results. C6orf118, CXorf58 and TMEM232 were proposed to be involved in cilia-related functions; TMEM53 and SMLR1 were proposed to be involved in lipid metabolism and C7orf25 and RSRP1 were proposed to be involved in RNA metabolism and gene expression. Experimental strategies to test these hypotheses were also discussed. The results of this manual data mining study may contribute to the project recently launched by the Human Proteome Organization (HUPO) Human Proteome Project aiming to fill gaps in the functional annotation of human proteins.

Database URL: http://www.nextprot.org

## Introduction

Biomedical research is an area generating a prodigious amount of information obtained from massive datasets. Databases organizing, standardizing and distributing this information, as well as bioinformatics tools allowing to mine these data, so as to translate it into knowledge useful for discovery are thus crucial to modern biological research. neXtProt is a knowledge platform that provides advanced query tools to explore the universe of human proteins (1). In neXtProt, ∼90% of the ∼20 000 predicted human protein coding genes have functional annotations derived from direct experimental characterization in human or model organisms or inferred by similarity to characterized proteins sharing sequence or structure similarities (1).

The HUPO Human Proteome Project (HPP) has recently launched a project aiming to characterize the remaining 10% of human proteins that have no annotated function (2). While lacking functional information, these proteins or their orthologs have associated information in neXtProt and other databases such as subcellular location, protein–protein interactions, tissue expression, post-translational modifications (PTMs), association with diseases and three-dimensional structure (3). In the past years, we have shown that it was possible to propose hypotheses for the function of such proteins by combining information from the literature, experimental repositories, databases and bioinformatics tools (3–5). These proposed functions have been published and are awaiting experimental validation to be annotated in neXtProt.

In order to accelerate the characterization of human proteins that have no annotated function and building on the experience we have acquired from our previous work, we developed a workflow to generate hypotheses for the function of these proteins based on a combination of annotations from biological databases and results from prediction tools. This workflow has been used to teach third-year undergraduate students in biomedical science at the University of Geneva how to use biological databases and bioinformatics tools and interpret the results. At this stage of their education, the students already have a good knowledge of biological concepts, allowing them to get involved in so-called course-based undergraduate research experiences (CUREs). In CUREs, students learn the scientific method (recognizing and formulating a research problem, collecting data, formulating hypotheses and testing them) by addressing a research question that is of interest to the scientific community (6). CUREs of different scales have been developed in biology and particularly in bioinformatics. For example, the SEA-PHAGES program (Science Education Alliance Phage Hunters Advancing Genomics and Evolutionary Science) incorporated 5000 undergraduates from different institutions to isolate, sequence and analyze hundreds of bacteriophage genomes (7). The Community Assessment of Community Annotation with Ontologies (CACAO), a competition for intercollegiate teams, incorporated nearly 800 undergraduates over a decade to generate about 5000 Gene Ontology (GO) annotations for species spanning all domains of life based on experimental observations published in the literature (8). Our course was tailored for a class of undergraduate students, typically 20 students in total, and will be repeated annually. The aim is not to produce annotations from published experiments as in CACAO but to propose functional predictions that will be testable in research laboratories. In that respect, it is more similar to another CURE that took place a few years ago and aimed to predict the function of uncharacterized open reading frames (ORFs) of *Saccharomyces cerevisiae* (9). Our course was named Functionathon because, like in a software development hackathon, students worked in teams to tackle a research problem. However, in contrast to hackathons that typically take place for a very limited time, this course lasted one semester, which is a more typical time frame for a bioinformatics research project. In this paper, we present the workflow, so that it can serve as a basis to organize similar courses at other universities, the results obtained by its application to the selected set of seven uncharacterized proteins and experimental strategies that could be used by the scientific community to test the hypotheses generated. These outcomes represent a direct contribution to the HPP, in terms of both methodology and results. We believe that the workflow presented will help any researcher with no prior expertise in biocuration or data mining who tries to predict the function of an uncharacterized protein and faces the diversity of databases and tools available. We hope the proposed hypotheses will foster collaborations within and beyond the HPP consortium and will soon be confirmed experimentally so that they can help fill gaps in the functional annotation of the human proteome.

## Methods

Initial literature searches were performed by the tutors in December 2019. The first draft of the identity cards of the candidates was built using UniProtKB (10) release 2019_11, neXtProt release 17 January 2020, and the versions available in January 2020 for all the databases and tools described in the next sections. Subsequent releases from February 2020 to February 2021 were used by the students and the tutors in order to update the information. The literature was also monitored during that time.

For all bioinformatics tools, default parameters were used unless specified otherwise. For neXtProt, only gold annotations were considered. The URLs for the databases and tools are recapitulated in Supplementary Table S1.

### Phylogenomic analysis

The presence of homologs for the selected proteins was checked in TreeFam (11), EggNOG (12) and The Orthologous Matrix (OMA) database (13).

BLASTp analysis was done at both NCBI and UniProt against all nonredundant sequences at NCBI (nr) and UniProtKB reference proteome + SwissProt at UniProtKB and set to retrieve the maximum of hits. Orthologs were inferred by reciprocal best hit BLASTp. psiBLAST was run against all nonredundant sequences at NCBI (nr) with a maximum of six iterations. tBLASTn at NCBI against nucleotide collection (nr/nt) limited to a specific taxon was used to search for sequences when not found by BLASTp or psiBLAST.

For HHPRED (14), we searched both against all proteomes and only the human proteome (Euk_Homo_sapiens_04_Jul_2017). For SWISS-MODEL (15), we used the automatic modeling option.

Multiple sequence alignments were done at EBI via the European Bioinformatics Institute servers with Clustalw Omega (16), colored with Jalview and manually annotated with PTM and domain information retrieved from UniProtKB/Swiss-Prot, neXtProt and InterPro (17).

### Sequence-based subcellular location prediction

For the human protein, subcellular location annotations were retrieved from neXtProt. For the orthologs in model organisms, predicted transmembrane domains and signal sequences were retrieved from UniProtKB.

As general subcellular location prediction programs, we used WoLF PSORT (selecting the corresponding organism: plant, animal or fungi) (18) and DeepLoc (hyperparameter by default: Profiles) (19). For WoLF PSORT, we reported subcellular location with scores ≥5 and for DeepLoc a likelihood ≥0.2.

Transmembrane domains were predicted using TMHMM2.0 (20) and Phobius (21). Mitochondrial transit peptides and signal peptides were predicted using TargetP2 (22), iPSORT (23) and SignalP (24), respectively.

MitoProt (25) was used for mitochondria prediction (cutoff 0.7) and SecretomeP-2.0 (26) for nonclassical secretion. NLStradamus (27) and seqNLS (28) were used to predict nuclear localization signals. Nuclear export signals were predicted with netNES (29) and LocNES (30). GPI-anchors were predicted by big-PI Predictor (31).

### Protein–protein interaction data

Protein–protein interaction information was retrieved from neXtProt that integrates human data from UniProtKB/Swiss-Prot, IntAct (32) and Enyo Pharma (http://www.enyopharma.com/). Only interactions confirmed with at least two experiments were considered.

### Expression and coexpression data analysis

Human expression data were first explored using the Human Protein Atlas (HPA, Version 19, RNA-seq and antibody-based data) (33). For antibody-based tissue expression data, only data with ‘enhanced’ validation were considered. For HPA RNA-seq data, we reported for each gene the tissue specificity annotation, based on normalized expression: tissue enriched, group enriched, enhanced, low specificity and not detected.

Human and mouse expression data were then explored using Genevestigator version 7.4.0 (mRNA-seq Ensembl 97 GRCh38.p12 and Affymetrix Human Genome U133 Plus 2.0 Array; Affymetrix Mouse Genome G430 2.0 array, mRNA-seq Ensembl 88 GRCm38p5) (34). Part of this database is accessible for academics. For both RNA-seq and microarray data, we reported the tissues in which high or medium expression was observed. Data obtained on organoid cultures and cells differentiated in *ex vivo* cultures, such as mesenchymal stem cell–derived adipocytes, were ignored. mRNA-seq gene expression in Genevestigator is measured in transcripts per million. For humans, high and medium expressions correspond to the log (2) of the average of the mean value higher than 3 and 0, respectively; for mouse, high and medium expressions correspond to the log (2) of the average of the mean value higher than 1.25 and 0, respectively. For microarrays, gene expression is expressed as log (2) relative mean values. For human and mouse, high and medium expressions correspond to values higher than 11.5 and 8.5, respectively.

Coexpression analysis for the human protein was performed using microarray data in Genevestigator version 7.4.0 and a combination of microarray and RNA-seq data in SEEK (35). For Genevestigator, the coexpression analysis (only available upon license or for teaching) was carried out in two steps. First, using the ‘perturbation’ tool, the studies in which the expression of the gene of interest was upregulated or downregulated were selected (fold change >1.5 and *P*-value 0.05). Then, using the ‘coexpression’ tool option anatomy, the list of the top 100 genes coexpressing with the uncharacterized gene was retrieved. For SEEK analysis, the top 100 genes were analyzed. Both Genevestigator and SEEK gene names were mapped to Swiss-Prot accession numbers using the ID mapping tool at the UniProtKB website, and the Swiss-Prot accession numbers were then used in the following step.

The list of genes obtained with Genevestigator (releases 10 August 2020 and 08 October 2019) and SEEK (release 24 February 2021) were analyzed with the statistic GO term overrepresentation analysis at PANTHER (36) using the annotation DATA SET ‘GO biological process complete’. Overrepresented GO terms/pathways were determined using default parameters (Fisher’s exact test, False Discovery Rate (FDR) *P* < 0.05). All the positive overrepresented GO biological process (BP) terms were considered, and the more precise GO terms selected using the hierarchical ontology view of PANTHER. The more specific GO terms were manually grouped into categories in order to summarize the data.

### Phenotypes and diseases

Phenotype information was retrieved from UniProtKB and the model organism databases for the organisms in which the protein is conserved: MGI (37), Zfin (38), Xenbase (39), Flybase (40), Wormbase (41), SGD and TAIR/Araport. This information was complemented with information from the literature and the International Mouse Phenotype Consortium (IMPC) (42).

Disease information was retrieved from neXtProt. Results from genome-wide association studies associating single-nucleotide polymorphisms with human diseases or traits were gathered from the literature.

### Ontologies

The GO (43) was browsed at QuickGO (44) and at AmiGO (45). The Evidence & Conclusion Ontology (ECO) (46) was searched at the EBI.

## Results and discussion

### The hypothesis generation workflow

**Figure 1.**
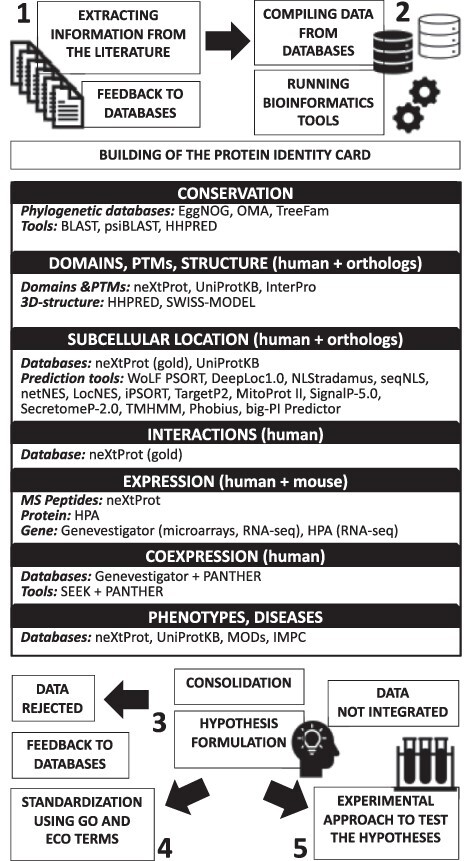
Schematic illustration of the proposed hypothesis generation workflow for uncharacterized human proteins.

As shown in Figure 1, our hypothesis generation workflow is divided into five tasks.

#### Extracting information from the literature

The function of newly characterized proteins, or their association with diseases, may not yet be annotated in databases. In addition, proteins that have been characterized but for which authors did not use the official gene name or for which the official gene name changed after publication are difficult to identify by curators. For these reasons, the official gene name and all the synonyms retrieved from neXtProt, UniProtKB and model organism databases were used to search in PubMed, PMC and Google to spot functional or clinically relevant data hidden in the literature. When a characterization study was spotted, it was sent to UniProtKB/Swiss-Prot to update the function of the protein.

#### Compiling the available data and running bioinformatics analysis tools to build the protein identity card

For proteins for which no characterization study has been found by extensive literature search, we explored the data available in public databases and ran bioinformatics analysis tools to predict function(s) and help in the design of experiments to validate these predictions. We used primary databases that produce their own data (e.g. HPA) and knowledge databases that manually integrate data from primary sources (e.g. UniProtKB, neXtProt and model organisms databases). Building the identity card of the protein of interest helps to organize the information relevant for formulating hypotheses and contains the following information: (i) official gene name, identifiers and synonyms; (ii) conservation; (iii) domains, PTMs and three-dimensional structure; (iv) subcellular location(s); (v) protein–protein interactions; (vi) expression and coexpression and (vii) phenotypes and diseases. In some cases, articles covering these aspects were found during the previous literature mining step. The information was noted in the corresponding section with its respective reference.

For a given topic, the available resources might have some degree of overlap in their content. As algorithms based on different properties and using different training sets may give different results, the same datasets can be annotated differently. In principle, all the data retrieved should be combined to maximize the information coverage, but in some cases, extracting the consensus from a selection of resources is required to strengthen its reliability. As the different databases share contents and sometimes independently annotate the same experiments, keeping track of the references for each annotation avoids reporting data as independent observations when they are based on the same observation.

Databases and tools are constantly evolving, and different versions may lead to different results. Hence, regularly updating the information using the most recent versions of resources is important.

##### Conservation

Study of the conservation of the protein allows potential paralogs and orthologs to be detected. When a paralog is found, the results of the subsequent analyses are compared with what is known about the paralog.

In addition, it enables the evolutionary history of the gene of interest to be constructed. The absence of the protein in certain branches or its presence in others can be indicators of specialized functions. For example, phylogenetic profiling has been successfully used to identify genes involved in ciliated functions (47).

Phylogenomic databases, BLAST, psiBLAST and HHPRED were used to look for homologs, with a focus on the model organisms *Mus musculus*, *Gallus gallus*, *Xenopus tropicalis*, *Xenopus laevis*, *Danio rerio*, *Drosophila melanogaster*, *Caenorhabditis elegans*, *S. cerevisiae* and *Arabidopsis thaliana* (listed in increasing evolutionary distance from man). We considered all the orthologs proposed by the different phylogenomic databases. Performing protein BLAST (BLASTp) and psiBLAST allows sequences that are absent in phylogenomic databases to be identified. Nucleotide BLAST (tBLASTn) at NCBI is used to systematically look for orthologs in the genomes of model organisms when they have not been found in databases or by BLASTp and psiBLAST. HHPRED is a highly sensitive method based on HMM–HMM alignments that allow to do structure prediction and to detect remote protein homologies where BLAST search fails.

The multiple alignment of homologous sequences covering different phylogenetic lineages—and at least the selected model organisms when they exist, helps to evaluate the quality of the selected sequences. For example, sequences in which the N-terminal or C-terminal regions are too long or too short may be indicators of wrong gene predictions and should be removed from the alignment and from further analysis. The multiple sequence alignment also allows conserved regions and sites to be detected.

##### Domains, PTMs and three-dimensional structure

Domains, binding sites and PTMs are important for protein function because they may be directly involved in function or regulate the activity of the protein or its interactions with other molecules. UniProtKB/Swiss-Prot contains experimental and predicted information about sites, domains and PTM for human and orthologs. neXtProt contains additional PTM information for the human proteins from Glyconnect, PeptideAtlas and its own annotations. InterPro contains additional information about domains integrated from various databases. SWISS-MODEL and HHPRED can help identify additional active sites or structural domains based on structural similarity. The available experimental or modeled three-dimensional structure of the protein can be used to check that predicted domains, PTMs and active sites are in agreement with the folding constraints and accessibility.

##### Subcellular location

Proteins must be targeted to the appropriate compartments to perform their functions. The different subcellular locations determine if the protein has access to metabolites and interacting partners.

Subcellular location annotations were first retrieved from UniProtKB and neXtProt. UniProtKB uses TMHMM, Phobius and SignalP to automatically annotate transmembrane domains and signal sequences. In UniProtKB/Swiss-Prot, these annotations are manually reviewed and enriched with other predictions, such as mitochondrial target peptides, and with experimental observations. For human proteins, neXtProt adds experimental information from GO and HPA. Wolf PSORT and DeepLoc, which are general subcellular location prediction programs based on different algorithms, were run for the human and model organism ortholog sequences for proteins without experimental subcellular localization in order to define their most probable localization(s) or to compare and predict the localization of orthologs. The cutoff to consider a prediction as significant or not is not clearly documented. For WolfPSort, we followed the same approach as other databases (48), and for DeepLoc, we took all the subcellular locations with a cutoff higher than 0.2, assuming that localizations that had 80% of chance to be correct among the 10 localizations tested were considered. Wolf PSORT and DeepLoc results were refined using more specific tools (e.g. nuclear, mitochondria, nonclassical secretion and GPI-anchor).

##### Protein–protein interactions

In cells, proteins rarely act in isolation but rely on a network of interactions with other proteins to perform their functions. Physical interactions found in low- or high-throughput experiments can be used to infer protein function. The known or predicted subcellular locations of the interacting partners indicate if these interactions may occur *in vivo*. Human protein–protein interactions were retrieved from neXtProt, together with information on the study in which these interactions were identified (PMID) and the experimental methods used. For each interactor, the function [gold molecular function (MF) and/or BP GO terms, pathways and free text overview]\, subcellular localizations and disease information annotated in neXtProt were analyzed and summarized.

##### Expression

The RNA-seq data that were mined were obtained on polyadenylated messenger RNA (mRNA) and not on total RNA, which increases the probability that genes with positive expression are protein coding. However, one cannot exclude that these transcripts may correspond to noncoding polyadenylated mRNAs (49). The definite proof that the genes are protein coding can only be derived from experiments analyzing the protein itself, e.g. with mass spectrometry-based techniques. neXtProt contains information about peptides identified in biological samples from the PeptideAtlas and MassIVE repositories and uses this information to validate the existence of human gene products.

Functional clues can be inferred if the expression of the gene is specific to a functional system, tissue, organ or cell type or enriched in a group of tissues or organs sharing a similar function. Whereas the mouse gene expression at the RNA level was only explored using Genevestigator, the human gene expression was explored using both Genevestigator and HPA, which are complementary in terms of contents and functionalities. These databases have manually curated microarrays (Genevestigator) and RNA-seq (HPA and Genevestigator) data from various tissues and cell types. Data are quantitatively displayed and organized in functional systems allowing the comparison between different anatomical structures.

Because protein and transcript abundances are not always well correlated, it is important to examine the expression profile of the gene at the protein level. HPA provides tissue expression profiles obtained by immunohistochemistry for human genes with validation scores. Expression profiles with an ‘enhanced’ score have been validated orthogonally with RNA-seq data or with a combination of antibodies.

Comparison of expression data in human and mouse indicates if the expression profile is conserved in these species and strengthens functional clues derived from expression data. Expression comparison is not a trivial analysis because the apparent lack of conservation can be the result of incomplete experimental data, similar functions performed by different organs in the different species or an indication of divergence in protein function.

##### Coexpression

Gene coexpression analysis is used to associate genes of unknown function with BPs. The results of coexpression are very sensitive to the methods and the parameters used; it is thus important to analyze coexpression with different programs that use different approaches. Regardless of the method, it is important to keep in mind that coexpression is the result of a correlation of expression at the mRNA level and does not necessarily reflect regulation at the protein level and does not provide information on causation or distinguish between regulated and regulatory genes. The output of a coexpression analysis is a list of genes that can be analyzed with an overrepresentation test to investigate if there is an enrichment in particular processes. This analysis is highly dependent on the degree of annotation of the genes under consideration. The use of PANTHER to analyze the list of coexpressed genes not only ensures that the annotations are up to date but also that the GO terms are displayed hierarchically, enabling the GO terms to be grouped by shared parent terms and the results to be summarized.

##### Phenotypes and diseases

Mutant phenotypes in model organisms may provide hints as to the function of the human protein. IMPC aims to create knockout strains for all mouse genes and characterize their phenotype through standardized protocols. It is particularly important to check the conditions in which the phenotypes were obtained (homozygous or heterozygous mutants, male or female animals, development stage, etc.), as subtle phenotypes may be revealed only under specific conditions.

#### Consolidating the data to formulate hypotheses

All the data gathered should be checked for inconsistencies, and potential conflicts should be carefully examined. This manual step is critical as it enables the coherence of the data compiled to be determined. At this stage, we do not advise to systematically prioritize experimental data versus predictions: predictions with good confidence scores may be more relevant than unreliable experimental data.

Functionally important sites, such as active sites or binding sites and domains, are expected to be conserved among a wide range of orthologs. PTMs, which generally have a regulatory role, might be less conserved, as the modification can be made in other nearby residues in the three-dimensional structure.

PTMs can influence the subcellular location of the protein. Conversely, a protein can only be post-translationally modified if it comes in contact with the enzyme catalyzing the modification and if the physicochemical conditions of the environment are appropriate. Therefore, any predicted or experimental PTM is checked for compatibility with the predicted or experimental subcellular location of the protein.

Similarly, the subcellular location of each interactor retrieved from databases is compared with the subcellular location of the protein of interest to check that the two proteins can interact *in vivo*.

In addition, it is interesting to know if the interacting partners coexpress with the uncharacterized gene. If the interacting proteins coexpress, this may indicate a close dependence for function or regulation.

Once the collected data have been checked, functional hypotheses can potentially be inferred. We define a functional hypothesis as an educated guess of the protein’s function based upon the elements of the identity cards combined with previous knowledge in the field. The predicted functions can be MFs (e.g. enzyme activity) or processes taking place at the level of the cell, system or organism but should be specific enough to allow scientists to design experiments to test them.

General rules for functional inference based on the collected data are difficult to establish. In general, sequence- or structure-based data, such as the presence of functional domains, might aid in building MF-based hypotheses. Phylogenomic profiling and data that reflect the spatiotemporal context of the protein, such as expression profile, protein–protein interactions or coexpression, are expected to generate BP-based hypotheses.

The hypotheses are refined until the predicted function(s) is/are compatible with the information on the protein and what is known in the field. For example, if a protein is proposed to play a key role in a system, then this system should be impaired in mutant animals. In these hypothesis generation and refinement tasks, it should be kept in mind that proteins can have several MFs and multiple roles in different BPs, depending on the tissue or the organism in which they are expressed, their interactors and subcellular location. If some of the data do not support the predicted functions, it should ideally trigger the generation of an additional prediction. Data that were not rejected but do not support any testable hypothesis was stored in the section ‘Additional comments’.

#### Standardizing hypotheses using GO and ECO terms

GO describes genes and their products in terms of MF, BP and cellular components and is the standard ontology to analyze and share data. Accordingly, proposed functions at the molecular level and at the level of a BP were expressed using MF and BP GO terms, respectively. These terms were searched by browsing the GO.

The evidence used to build these hypotheses, originating from experiments, computational methods or literature curation, were described using terms from the ECO.

In both GO and ECO ontologies, the most specific terms were chosen. The selection was based on the definition of the term, rather than the term name, to ensure that the concept is correctly attributed.

#### Proposing an experimental approach to test the predictions

Because scientific hypotheses must be testable, the workflow includes looking for possible experimental approaches to test the predicted functions.

In order to provide the basis for further functional characterization, the key data used to build the functional hypotheses should be confirmed, as they often result from high-throughput experiments. Antibody-based techniques such as immunofluorescence, immunohistochemistry or co-immunoprecipitation can be used to validate expression, subcellular location and interaction data. HPA is an invaluable source of antibodies that are provided with a detailed description of their suitability to different techniques.

The strategy to validate a functional prediction will depend on the function(s) to be tested and will preferably be based on a combination of orthogonal methods.

Recombinant proteins can be used to test MF predictions. Subcellular and PTM analysis may guide the choice of the system to express the protein. For example, secreted proteins should be expressed in eukaryotic systems and collected in the extracellular medium in order to respect their folding (disulfide bonds) or PTMs (glycosylation or cleavage).

Human cell lines are routinely used for preliminary characterization studies as they are easy to manipulate, inexpensive and minimize ethical issues. HPA contains expression data at mRNA and protein levels on a broad panel of 63 cell lines that can be used to identify which cell line expresses the gene or protein of interest. The main drawback is that most cell lines are cancer cell lines that might not completely reproduce the physiology of normal cells. Functions that are specific to differentiated cells are difficult to study in cell lines and should instead be studied in primary cells or induced stem cells. Biological processes that involve a dialog between different cell types, such as immune response, development or reproduction, need more sophisticated systems such as organoid cultures or *in vivo* models.

The choice of the animal model will not only depend on the conservation profile of the protein but also on the adequation between its anatomical, physiological or behavioral properties and the function to be studied. As a general principle, the closer the system resembles that in humans, the better because results can be extrapolated to humans with more confidence, but for ethical reasons, it is better to use evolutionary distant organisms. Mouse is by far the most common mammalian model. Available mouse embryonic stem (ES) cells and mutant animals are available from the International Mouse Strain Resource. In many cases, available ES cell clones have been targeted using the ‘knockout-first, conditional-ready’ approach (50). This approach allows flexibility in the design of the knockout strains and allows the gene deletion to be restricted in a tissue- or time-specific manner by breeding mice with specialized Cre-Lox and FLP-FRT recombination systems. IMPC null allele mice strains used in the primary phenotyping pipeline and conditional-ready allele strains are also available. Mutant strains in other vertebrate species are also available in the model organism databases (Zfin and XenBase).

Regardless of the cellular or animal model chosen, the strategy usually consists in downregulating the expression of the considered gene by genome editing and measuring phenotypes at the cellular or organism level. This strategy can fail when another gene can substitute for the targeted gene, preventing the observation of any phenotype. In this case, comparison between single and double mutants and complementation analysis may help to elucidate their function.

This approach can also be difficult if the targeted gene has a function that cannot be easily studied in the usual standardized settings of a laboratory (e.g. response to pathogens or environmental conditions). In those cases, the phenotyping needs to be done in optimized conditions.

### Application of the workflow to seven uncharacterized proteins in the frame of the Functionathon course for undergraduates

#### The Functionathon course

The workflow, which has been designed for scientists without experience or competencies in bioinformatics, has been tested with a class of undergraduate students in biomedical sciences.

The tutors selected seven proteins for which no experimental characterization had been published in the literature, and evidence of expression at the transcript level was available. They ensured that these proteins were conserved outside primates to increase the probability of finding experimental data in model organisms and checked that high confidence data were available for at least two of these sections of the ID cards: domains and/or PTM, interactions, subcellular location, phenotypes and/or diseases. This was done in order to guarantee that the students would be able to explore and integrate these different aspects together with conservation, expression and coexpression data.

The 20 students were randomly distributed into seven groups. Each group was randomly assigned one of the seven selected uncharacterized human proteins. During six practical sessions of 3 hours each, the students followed the workflow described above in order to gather, critically analyze and synthesize the information. Sessions started with a brief introduction on how to use the relevant tools and interpret the data, and the rest of the time was dedicated to the collection of data. In order to guide the students, a list of the objectives and mandatory tasks to achieve were provided at each session. Each group was assigned to one tutor, who was available to answer questions and discuss the work shared using online documents.

By comparing the collected data to their own data mining results, tutors ensured that students were able to find all the relevant information, organize their findings and discuss conflicting data.

The data integration and hypothesis formulation require time to study or review the BPs that are the output of the coexpression analysis, that describe the function of the interacting proteins and that can be associated with the subcellular location(s) of the protein and their interactors. These steps largely depend on the amount of the gathered data and the biological background of the students. Tutors helped the students by answering their questions and providing informative resources on specific biological concepts.

The definition of an experimental strategy to validate the hypothesis requires some experience with laboratory techniques. Although the students had a good theoretical biological background, they obviously lacked such expertise. Tutors helped them to design and refine the experimental validation strategies. At the end of the course, the students reported their research results in the form of an oral presentation in which each member of the group participated. In addition to the tutor, four senior scientists independently evaluated the presentations and provided some feedback about the proposed experimental strategies. The consensus among the evaluators was that all the presentations were of high quality and that students had not only acquired bioinformatics knowledge but also communication skills and scientific maturity. During the final discussions, some students suggested drafting a publication with the data mining, the functional hypotheses proposed and ideas for experimental verification. This suggestion confirmed that the students projected themselves as real researchers in this project. As this coincided with the end of the academic year and their undergraduate curriculum, we undertook the writing of this manuscript.

The standardization of functional hypotheses using GO/ECO terms was performed by the tutors after the course, as assigning GO and ECO terms requires special training that was out of scope for the course.

#### Application of the workflow to the seven proteins

The identity cards obtained for the seven uncharacterized proteins are shown in Supplementary File S1, as well as the reasoning that led us to predict function(s) for these proteins and additional comments. Details on subcellular information and expression are provided in Supplementary Tables S2 and S3. Details on coexpression analysis using Genevestigator and SEEK are provided in Supplementary Tables S4 and S5. Multiple alignments are provided in Supplementary File S2 as annotated figures and in Supplementary File S3 in FASTA format.

#### Standardization of the hypotheses using GO and ECO terms

In contrast to function prediction methods based solely on sequence or structure similarity search which are more prone to predict MF than BP GO terms (51), our workflow integrates phylogeny, coexpression, protein–protein interaction, subcellular location, phenotype and disease data and can also predict GO BP terms. Table 1 recapitulates the predicted GO MF/BP terms for the seven proteins, with their evidence expressed as ECO terms. Out of the 18 unique GO terms predicted, only 3 are MF GO terms (RNA binding, ribonuclease activity and lipase activity). As expected, those were predicted based on the presence of domains (ECO:0000260), on positional similarity evidence (ECO:0007094) or on structural similarity evidence (ECO:0007090). The GO BP terms were predicted based on phylogeny (ECO:0007153), expression (ECO:0000270), coexpression (ECO:0007099), subcellular location (ECO:0000087), protein–protein interaction (ECO:0000353) and disease association (ECO:0001237) data. Phenotype and PTM data could not be used directly to predict function but were checked for compatibility with the proposed hypotheses and used to define the experimental validation strategy.

**Table 1.** Predicted functions for the seven proteins, expressed as GO MF/BP terms with their evidence expressed as ECO terms.

**Gene name**	**neXtProt AC**	**Chr**	**Predicted functions (GO terms)**	**Evidence (ECO terms)**
**C6orf118**	NX_Q5T5N4	**6**	1—Determination of left–right anatomical asymmetry (GO:0007368)	Phylogenetic distribution evidence used in manual assertion ECO:0007153 (1–3)
			2—Motile cilium assembly (GO:0044458)	Gene expression similarity evidence used in manual assertion ECO:0007099 (1–4)
			3—Cilium movement (GO:0003341)	Expression pattern evidence used in manual assertion ECO:0000270 (1–3)
			4—Protein localization to cilium (GO:0061512)	Natural variation mutant evidence used in manual assertion ECO:0001237 (2, 3)
**CXorf58**	NX_Q96LI9	**X**	1—Motile cilium assembly (GO:0044458)	Phylogenetic distribution evidence used in manual assertion ECO:0007153 (1–3)
			2—Cilium movement (GO:0003341)	Gene expression similarity evidence used in manual assertion ECO:0007099 (1–3)
			3—Spermatogenesis (GO:0007283)	Expression pattern evidence used in manual assertion ECO:0000270 (1–3)
**TMEM232**	NX_C9JQI7	**5**	1—Non-motile cilium assembly (GO:1905515)	Gene expression similarity evidence used in manual assertion ECO:0007099 (1–4)
			2—Motile cilium assembly (GO:0044458)	Expression pattern evidence used in manual assertion ECO:0000270 (1–3)
			3—Cilium movement (GO:0003341)	Natural variation mutant evidence used in manual assertion ECO:0001237 (2, 3)
			4—Protein localization to cilium (GO:0061512)	
**C7orf25**	NX_Q9BPX7	**7**	1—Ribonuclease activity (GO:0004540) [MF]	Match to InterPro member signature evidence used in manual assertion ECO:0000260 (1, 2)
			2—RNA binding (GO:0003723) [MF]	Gene expression similarity evidence used in manual assertion ECO:0007099 (3, 4)
			3—DNA-dependent transcription (GO:0006351)	Physical interaction evidence used in manual assertion ECO:0000353 (4–6)
			4—mRNA metabolic process (GO:0016071)	
			5—tRNA metabolic process (GO:0006399)	
			6—Translation (GO:0006412)	
**RSRP1**	NX_Q9BUV0	**1**	1—mRNA splicing via spliceosome (GO:0000398)	Gene expression similarity evidence used in manual assertion ECO:0007099 (1, 3)
			2—Regulation of cell cycle (GO:0051726)	Physical interaction evidence used in manual assertion ECO:0000353 (1–3)
			3—Regulation of transcription by RNA polymerase II (GO:0006357)	Expression pattern evidence used in manual assertion ECO:0000270 (2)
**SMLR1**	NX_H3BR10	**6**	1—Lipid metabolic process (GO:0006629)	Gene expression similarity evidence used in manual assertion ECO:0007099 (1, 2)
			2—Lipid homeostasis (GO:0055088)	Expression pattern evidence used in manual assertion ECO:0000270 (1, 2)
				Physical interaction evidence used in manual assertion ECO:0000353 (1)
**TMEM53**	NX_Q6P2H8	**1**	1—Lipase activity (GO:0016298) [MF]	Match to InterPro member signature evidence used in manual assertion ECO:0000260 (1)
			2—Lipid metabolic process (GO:0006629)	Structural similarity evidence used in manual assertion ECO:0007090 (1)
				Positional similarity evidence used in manual assertion ECO:0007094 (1)
				Gene expression similarity evidence used in manual assertion ECO:0007099 (2)
				Immunolocalization evidence ECO:0000087 (2)

Based on the predicted GO terms, the seven proteins can be associated with three high-level functional categories: C6orf118, CXorf58 and TMEM232 with cilia-related functions, C7orf25 and RSRP1 with RNA metabolism and gene expression and TMEM53 and SMLR1 with lipid metabolism.

#### Proposed validation approaches

The experimental strategies proposed to validate our hypotheses in each functional category are based on the available tools for each protein (antibodies and knockout mice), recapitulated in Supplementary Table S6, and on the CRISPR/Cas9 technology that can be used to edit genes within cells and organisms (52).

##### Cilia-related functions of C6orf118, CXorf58 and TMEM232

In contrast to C6orf118 and TMEM232, CXorf58 awaits validation at the protein level, according to neXtProt. Antibody- or mass-spectrometry-based experiments should be planned to ensure that this gene is protein coding.

Multiciliated cells generated from human-induced pluripotent stem cells (53) or human primary nasal airway epithelial cells (54) could be used to study the function of C6orf118, CXorf58/MFI and TMEM232 in motile cilia assembly or function. A possible function of TMEM232 in non-motile cilia assembly could be tested by downregulation approaches in RPTEC/TERT1 cells (55).

Knockout mice are available for C6orf118 and Tmem232. Non-motile (sensory) cilia-associated phenotypes that could be measured are defects in hearing, vision and limb patterning. Motile cilia-associated phenotypes that could be measured are defects in lung clearance, establishment of left–right patterning during development, male and female fertility and cerebrospinal fluid circulation (56). In addition, Tmem232 knockout mice sensitized against allergens such as ovalbumin or pollen could be used to study a possible involvement of TMEM232 in cilia dysfunction associated with allergic rhinitis.

The potential role of CXorf58 in flagellar motility and spermatogenesis can be analyzed by generating inducible or spermatocyte-specific knockout mice (57, 58). As the mouse CXorf58 gene is overlapping with the FAM90a1b gene, knockout mice should be carefully designed by targeting the first exon.

A possible role for C6orf118, CXorf58/MFI and TMEM232 in ciliary processes can be studied in *Xenopus* and *D. rerio* after morpholino-based knockdown, CRISPR knockout or mRNA-induced overexpression. In *Xenopus* embryos, cilium movement can be measured in skin multiciliated cells (59). *X. laevis* can also be used to specifically study the role of C6orf118 in nodal cilia by live cell imaging of the fluid flow or cilia movement in the gastrocoel roof-plate, an epithelium homologous to the embryonic node (60). In *D. rerio*, the cilia function can be analyzed by measuring curving of the body axes, otolith formation perturbation, pronephric cyst formation or left–right asymmetry defects. This approach was used for MFI, the paralog of CXorf58 (61), and phenotypes obtained after CXorf58 and MFI downregulation should be compared. CXorf58 could have a redundant/overlapping function with MFI that would hinder the phenotype analysis. For all the proposed experiments in the different models, the effect of concomitant MFI and CXorf58 knockdown/knockout, as well as reciprocal phenotype rescue experiments, should be tested.

##### Functions of C7orf25 and RSRP1 in RNA metabolism and gene expression

The nuclease activity and substrate preferences of the recombinant human C7orf25 protein produced in *Escherichiacoli* can be studied using a fluorogenic assay (62).

C7orf25 has been shown to be expressed at fairly high levels and to be phosphorylated in HEK293 cells (63). These cells, which do not have the drawback of being cancer cells, could be used to analyze the effect of C7orf25 depletion on transfer RNA (tRNA) and mRNA levels and to test mRNA stability (64). Function in translation could be investigated by making polysome profiles (65).

RSRP1 expression is highest in cancer cell lines of lymphoid origin such as Karpas-707. These cells could be used to analyze the effect of RSRP1 depletion on the splicing of reporter genes (66) during the cell cycle, the transcription by RNA polymerase II (67) and the cell cycle progression.

The role of PTMs in the regulation of the function of both proteins could be analyzed by testing if mutants obtained by site-directed mutagenesis are able to rescue the phenotypes observed in knockout cells.

##### Lipid metabolic functions of SMLR1 and TMEM53

Human TMEM53 wild-type and site-directed mutants (Ser113Ala, Asp220Ala and His252Ala) could be expressed in *E.coli* and the lipase activity of these recombinant proteins analyzed with libraries of fluorogenic or chromogenic lipids of different sizes. Docking experiments could try to address if the predicted binding pocket can accommodate alkyl ester substrates and guide the choice of the substrates in the lipase assays.

HepG2 cells are liver cancer cells that express SMLR1 and TMEM53 at high levels. These cells could be used to analyze the difference in content or quality of lipids after downregulation of these genes, by a metabolomic approach. It would be informative to analyze if TMEM53 mutants of the proposed active site are able to rescue a wild-type phenotype in TMEM53 knockout cell lines. The interaction between SMLR1 and BSCL2 and the colocalization of the two proteins in lipid droplets and ER could be tested in those cells using the available antibodies from HPA, HPA066060 for SMLR1 and HPA042394 for BSCL2.

Available TMEM53 knockout mice (68) or generated using available ES cells could be used to analyze lipid levels and body mass. *D. rerio, X. laevis* and/or *M. musculus* SMLR1 mutants could be designed to analyze lipid levels and body mass.

#### Lessons learned from the Functionathon course

Extracting the relevant data from publications at the beginning of the workflow was found to be time-consuming. First, dozens of publications may be available for a single protein, even if it is uncharacterized. Then, the data often appear both in the main text and in supplementary data. Finally, the relevance of the information is only evident once the hypothesis starts to become evident, making it difficult for students to decide *a priori* how much time to spend on each article.

An important aspect of the data gathering step is to properly document the evidence of the information found in databases. Two main issues were identified when the students tried to report evidence for annotations. Firstly, some annotations were redundant. For example, neXtProt sometimes integrates the same subcellular localization data from HPA or UniProtKB and from GOA, as GOA also annotates subcellular location based on HPA and UniProtKB information. Secondly, annotations based on similarity with other proteins are difficult to trace as an explicit link to the similar sequence considered, its annotation and associated evidence is often missing. For example, in neXtProt and UniProtKB, a number of GO annotations inferred from the biological aspect of ancestor evidence with source GO_central are only associated with a publication describing how GO uses phylogenetic context to infer protein function (69), and the relevant information is only traceable from QuickGO at the EBI or AMIGO in the GO website. Finally, students faced the complexity of databases not only because the different databases have both different and shared data but also because they are differently structured.

The tutors identified a mistake in mouse genome databases (fam90a1b wrongly annotated as a CXorf58 ortholog) and one in the literature (TMEM232 wrongly described as a tetraspan protein) that would have been difficult for the students to spot. While MGI was notified and the mistake will be corrected, mistakes in the literature are generally not corrected.

One of the difficulties faced by students when they used bioinformatics tools was to establish cutoff and rules to consider a result as significant (e.g. Wolf PSORT and DeepLoc, BLAST, PANTHER and coexpression from Genevestigator). These difficulties are encountered also by experienced researchers as mentioned by Rafi *et al.*, who discuss that there is not a unique valid way to analyze data and highlight the importance of analyzing the information taking into account the context and the methods used to produce it. In addition, the authors of this study provide some clues for the proper interpretation of statistical tests (70).

The analysis of the coexpression results was not straightforward. Indeed, the output of the overrepresentation test obtained using PANTHER is usually many different BPs that are difficult to summarize in one or few pathways. In order to improve the precision of coexpression results, analyses based on RNA-seq data could be included. Comparison with mouse or other model organisms could also be useful. Microarray and RNA-seq data are available for mouse at Genevestigator.

The first experiments the students proposed were experiments to validate the experimental or predicted data collected, which is a good practice. Concerning the functional validation, the details to which students proposed experiments greatly depended on the knowledge the students had on the different subjects. Experiments to validate general BPs, such as transcription or translation, were more easily described than those concerning processes that are less discussed during their studies, such as ciliogenesis.

For the seven examples in this study, our workflow retrieved sufficient information for each of the human proteins to build testable hypotheses. However, in some cases, data may be scarce and additional resources or tools will need to be used to compile sufficient data to come up with a hypothesis. Additional high confidence genetic or physical interactions can be retrieved from databases such as BioGRID. STRING also provides predicted associations deriving from genomic context (gene fusions and neighborhood information). These associations can be used to infer function, as fused genes or neighboring genes may function in the same pathways. STRING also allows the user to perform a COG (clusters of orthologous groups of proteins) centric analysis to search for genes with the same or similar phylogenetic profiles. This tool could be useful for gene function prediction particularly if combined with the other options offered. Finally, manually curated GWAS databases such as GWAS Catalog (71) and GWAS ATLAS (72) could be used to fetch variants with the description of the associated diseases or phenotypes.

## Conclusion and perspectives

The primary output of this study is a data mining workflow that uses a combination of databases and tools to predict the function of uncharacterized human proteins. The final output of the workflow is predictions expressed as combinations of GO MF/BP and ECO terms and complemented by suggestions for experimental validation.

The workflow has been tested with undergraduate students on seven preselected proteins. Compared to the CACAO project, which is conceived as a competition, the Functionathon course is a collaborative project. Tutors give feedback all along the course and build functional predictions together with the students, which is closer to the situation in which master’s or PhD students work. While CACAO instructors can evaluate if the experimental data presented in the literature are correctly interpreted and translated into GO/ECO terms, tutors of CUREs like the Functionathon or the Yeast ORFan Gene Project (9) cannot themselves evaluate if the function predictions are correct: predictions will only be proven to be true or false after experimental validation by other researchers. One of the reported outcomes of the Yeast orphan gene research project was that students tended to be anxious to see whether they were ‘right’ in their hypotheses (9). Both tutors and students of the Functionathon shared similar concerns.

The Functionathon course can be adapted to master’s and PhD students that are able to work in a more autonomous manner. At that stage, students have a deeper and broader biological knowledge, may already have acquired some expertise at the laboratory (designing, planning and performing experiments) and experience in research projects. This background would allow them to formulate more precise hypotheses and experimental validation strategies. We hope that this study will trigger the creation of courses at different levels that will use our workflow and propose functions on a higher number of proteins. The workflow could potentially be applied to any randomly selected human uncharacterized protein; however, it should be kept in mind that it might be impossible to formulate a testable hypothesis if there are very little data or data that the researcher is not able to integrate. In such cases, we suggest that the researcher should try using complementary resources. Feedback and suggestions for improvements from students, researchers and teachers who will use the workflow are welcome.

For teaching purposes, we estimate that the workflow is appropriate because it involves interacting with the databases and tools in a thorough manner. The workflow is entirely manual and hence time-consuming. Automating some tasks could improve efficiency. The first task that could be automated is literature mining. Even if their function is still unknown, some proteins are associated with dozens of publications. Text-mining tools that allow the sentence in which the name of the protein appears together with the title of the study to be automatically extracted would accelerate the first step of the workflow. Database querying could also be automated. For example, all the gold data for one or several uncharacterized proteins and their interactors can be automatically retrieved from neXtProt in different formats using a combination of SPARQL is a recursive acronym for SPARQL Protocol and RDF Query Language, a semantic query language able to retrieve and manipulate data stored in Resource Description Framework (RDF) format SPARQL queries. Phylogeny data from OMA and UniProtKB annotations for ortholog proteins can also be extracted using SPARQL queries on their respective end points. Writing SPARQL queries requires some training, and tutorials have been recently published by neXtProt (73) and OMA (74).

Many function prediction tools based on machine learning and sequence alignment are under assessment by the Critical Assessment of Functional Annotation (75). The precision reached by these tools is still low (<0.5), even if some of them include protein-protein interaction data and the GO terms associated with interactors. The inclusion of various sources of data such as coexpression data may lead to improvements (76). It would be interesting to compare the results of computational methods with the results of manual data mining to evaluate if both approaches can complement or enrich each other. This comparison will be facilitated by the fact that both computational and manual predictions are expressed as GO MF/BP terms, but it will not be trivial because proteins can have several, nonmutually exclusive functions and because similar functions can be described with terms with variable precision (77).

neXtProt has just launched new community pages to host predicted functions. They have already been populated with the predictions generated in the present study (https://www.nextprot.org/entry/NX_Q9BPX7/function-predictions) and in a previous publication (3). They will be enriched with functional predictions from other publications (4, 5) and from the next editions of the Functionathon course. The pages will also be progressively populated with predictions from neXtProt users submitted via a dedicated form. The authors of the predictions will be credited through their ORCID identifiers. Converting free text into GO MF/BP and ECO terms requires specific training (78), and we encourage researchers to follow some of the tutorials available on the EBI website. Providing functional predictions based on carefully mined biological data along with proposed experiments to validate them will hopefully accelerate the characterization of poorly known proteins. Indeed, having a functional hypothesis to test generated by manual interpretation of existing data may be an advantage compared to starting a characterization project without a clear hypothesis to test. However, we cannot guarantee that these predictions will be confirmed as they may have been built on the basis of incomplete data or wrong interpretations. The neXtProt community pages show the standardized function predictions but not the detailed information underlying those nor the proposed validation experiments. Researchers using these predictions are thus encouraged to contact the submitters to get access to this information. We hope that this first contact will lead to fruitful collaborations between submitters of functional predictions and experimental researchers.

We hope that the current study will stimulate experimental studies on C6orf118, CXorf58 and TMEM232 in cilia-related functions and on TMEM53 and SMLR1 in lipid metabolism by the HPP teams who have demonstrated their expertise in characterizing human proteins involved in male reproduction (79), ciliogenesis (80) or liver metabolism (81). Researchers from the RNA biology community are welcome to join HPP and test the putative role of C7orf25 and RSRP1 in RNA metabolism and gene expression.

## Supplementary Material

baab046_Supp
