# Supplementary material for: Functionathon: a manual data mining workflow to generate functional hypotheses for uncharacterized human proteins and its application by undergraduate students
Source: Database (Oxford). 2021 Jul 28;2021:baab046. doi: 10.1093/database/baab046 (PMC8317215; doi:10.1093/database/baab046)
Supplement: baab046_Supp [file baab046_supp.zip › Legends for Supp Files.docx]

**Supplementary Table 1: List of all databases and tools used in the workflow, with their corresponding URL**

**Supplementary Table 2: Subcellular location**

**Column A:** gene name of the uncharacterized protein and the corresponding model organism. **Column B:** accession number corresponding to the sequence that was analysed. Whenever possible the UniProtKB accession number is indicated, otherwise RefSeq identifiers are provided. **Columns C and D** show the output of WoLF PSORT (scores >=5) and DeepLoc1.0 (likelihood >=0.2). The aberrant plastid predictions for animal proteins are highlighted in yellow. **Columns E to H** show the NLS and NES predicted by NLStradamus, seqNLS, netNES and LocNES. No indicates that no NLS, NES or GPI-anchor was predicted using the default parameters, and NA indicates that the analysis has not been done. The aberrant very long NLS preducted by NLStradamus are highlighted in yellow. **Columns I to O** indicate the output of PSORT II, TargetP2, MitoProt II, SignalP-5.0, SecretomeP-2.0, TMHMM, Phobius and big-PI Predictor. TM is for transmembrane domain, Yes indicates positive prediction, no indicates feature/subcellular location not predicted and NA indicates not analyzed.

**Supplementary Table 3: Expression in human and mouse**

Microarray (Affymetrix Human Genome U133 Plus 2.0 Array and Affymetrix Mouse Genome G430 2.0 array) and mRNA-seq (human Ensembl 97 GRCh38.p12 and mouse Ensembl 88 GRCm38p5) expression data for the seven human uncharacterized proteins and the mouse orthologs as provided by Genevestigator. Note that no data is provided for the mouse ortholog of Cxorf58 due to uncertain mapping. **Column A:** hierarchical path of the anatomical term. **Column B:** organ, tissue or cell type. **Column C:** additional description of the sample. **Column D:** number of samples used to annotate the expression. **Column E to K:** quantitative expression data as result of Genevestigator meta-analysis.

**Supplementary Table 4: Coexpression analysis with Genevestigator**

**Table 1:** List of genes coexpressing with the uncharacterized proteins according to Genevestigator analysis. For each uncharacterized protein the first column (column A, D, J, M, P, S) corresponds to the list of genes provided by Genevestigator and the second and third columns (columns B-C, E-F, H-I, K-L, N-O, Q-R, T-U) contain the mapping of this list to Swiss-Prot accession numbers. Between brackets are indicated the range of Pearson's correlation scores. **Other tables:** GO biological process overrepresentation analysis done with PANTHER on the coexpressed genes obtained with Genevestigator analysis. The column "Category" indicates the GO Biological Process term grouping done in order to summarize the results. Dark grey indicates the specific terms selected according to GO hierarchy.

**Supplementary Table 5: Coexpression analysis with SEEK**

Tables 1 to 6 contain the coexpression analysis done for six of the seven uncharacterized proteins. Note that SMRL1 is absent in SEEK platform. **Columns A to F**: output of SEEK analysis. **Columns H and I**: mapping of gene names to Swiss-Prot accession numbers. **Columns K to R**: Panther analysis. **Column S:** GO Biological Process term grouping done in order to summarize the results. Dark grey indicates the specific terms selected according to GO hierarchy.

**Supplementary Table 6: Antibodies available from HPA and ES cells or animals from the IMSR. IMPC production status is indicated.**

**Supplementary File S1: Identity cards and conclusions for C6orf118, C7orf25, CXorf58, RSRP1, SMLR1, TMEM53 and TMEM232**

**Supplementary File S2: Alignments for C6orf118, C7orf25, CXorf58, RSRP1, SMLR1, TMEM53 and TMEM232 and their corresponding legends.**

**Supplementary File S3: Alignments for C6orf118, C7orf25, CXorf58, RSRP1, SMLR1, TMEM53 and TMEM232 (FASTA format)**
