# Supplementary material for: Functionathon: a manual data mining workflow to generate functional hypotheses for uncharacterized human proteins and its application by undergraduate students
Source: Database (Oxford). 2021 Jul 28;2021:baab046. doi: 10.1093/database/baab046 (PMC8317215; doi:10.1093/database/baab046)
Supplement: baab046_Supp [file baab046_supp.zip › Supp_File_S1.pdf]

UNIPROTKB ACCESSION: Q5T5N4
GENE/PROTEIN NAMES: C6ORF118

CONSERVATION

Metazoa excluding Nematoda and Arthropoda.

MODEL ORGANISMS

M. musculus	1700010I14Rik/Q3TTN6
X. tropicalis	F6QEB3
X. laevis	A0A1L8G278
D. rerio	absent
G. gallus	truncated form

DOMAINS AND PTMS (HUMAN AND ORTHOLOGS)

- **InterPro: IPR032755:** Translin-associated factor X-interacting protein 1, N-terminal domain.
- **UniProtKB:** One or **two coiled-coil domains**. When two coiled-coil domains are annotated, the first one overlaps with IPR032755.

SUBCELLULAR LOCATION (HUMAN AND MODEL ORGANISMS)

- **neXtProt and UniProtKB:** no annotation.
- **WoLF PSORT and DeepLoc1.0:** cytoplasm then nucleus.
- **NLS and NES predictors:** no consensus among orthologs.

INTERACTIONS (HUMAN)

NextProt: None of high quality.

EXPRESSION (HUMAN AND MOUSE)

neXtProt: Validated at protein level: several unique peptides detected by mass spectrometry in male and female reproductive tissues.

LITERATURE

Enriched in several ciliary tissues in human (1) and mouse (2); induced during ciliogenesis in human (3).
Conflicting results in human neural progenitor cells infected with different strains of Zika virus: downregulated in some (4,5) but not all (6) studies. These differences do not seem to be due to the use of different virus strains or cell models.

HPA

No high quality data at the protein level.
RNA-seq: enhanced in brain, fallopian tube, pituitary gland and testis.

GENEVESTIGATOR

- **Human microarrays:** high in respiratory epithelium. Medium in all the systems.
- **Human RNA-seq:** high in respiratory epithelium, fallopian tube, fetal glia and neural progenitor cells, testis, adenohypophysis and pituitary gland. Medium in respiratory, nervous and sensory, embryonic and reproductive systems.
- **Mouse microarrays:** high in spermatogenic cells, testis and oocyte. Medium in most systems.
- **Mouse RNA-seq:** detected at medium and high levels in most systems. 30 times higher in testis than in the rest of the tissues.

COEXPRESSION (HUMAN)

GENEVESTIGATOR

cilium assembly, cilium movement protein localization to cilium.

SEEK

cilium assembly, cilium movement and spermatogenesis.

PHENOTYPES/DISEASES (HUMAN AN ORTHOLOGS)

- rs9365798 (intergenic SNP downstream of C6orf118): pulmonary tuberculosis risk in a Korean population (7)
- rs2675724 (127 kb downstream from C6orf118): asthma risk in a Latino female population (8).
- Knock-out mice: no significant phenotypes on mortality, ageing, growth, size, homeostasis, metabolism, adipose tissue, skeleton, limbs, digits, tail, craniofacial, hearing, cardiovascular and reproductive system (homozygous females and males) (IMPC).

PREDICTED FUNCTIONS

The enrichment in tissues with motile cilia in human and M. musculus (1-3), the coexpression with ciliary proteins (9), the association with a higher risk for asthma (8) and pulmonary tuberculosis (7, 10), conditions in which mucociliary clearance is impaired (11), and the phylogenetic profile mirroring the evolution of cilia (12), suggest that C6orf118 is involved in cilia related events. The absence of C6orf118 orthologs in Nematoda and Arthropoda, that only develop sensory/non-motile cilia, indicates that C6orf118 may be involved **in motile cilia assembly or function**. The enriched expression in multiciliated cells from respiratory epithelium indicates a function of C6orf118 in multiciliated cells. The absence in D. rerio and G. gallus, that do not have nodal cilia (13, 14) suggests that C6orf118 may also play a role in nodal cilia, which are motile monocilia responsible for generating the leftward flow of extracellular fluid that determines **left-right anatomical asymmetry** during development. In both mono and multiciliated cells, C6orf118 may be involved in **protein localization to cilium**, which may be important for ciliogenesis, cilium movement, or cilium signaling events. Despite its high expression in mouse testis, C6orf118 is probably dispensable for sperm flagella assembly as C6orf118 mouse mutants have a normal male fertility. The downregulation of C6orf118 in neural progenitor cells upon Zika virus infection in some experiments (4, 5) but not in others (6) deserves further investigation because it may be related to cilia dysfunction. Microcephaly induced by Zika virus infection during early pregnancy is associated with motile cilia dysfunction in ependymal cells (15).

ADDITIONAL COMMENTS

The domain IPR032755 present in C6orf118 is also found in two poorly characterized proteins: CLHC1, and TSNAXIP1. TSNAXIP1 interacts with TSNAX (16), which participates in several nucleic acid processing pathways including DNA damage response, microRNA degradation during synaptic stimulation, mRNA regulation during spermatogenesis. The subcellular location prediction programs and the comparison among orthologs suggest that C6orf118 is a cytoplasmic protein. The nucleus is predicted as a secondary localization, and a NLS is predicted only for the human sequence. Based on these observations C6orf118 may function in the nucleus in nucleic acid related functions. However, we are not able to propose a precise function to be tested experimentally.

REFERENCES

1. Ivliev, A. E., ’t Hoen, P. A. C., van Roon-Mom, W. M. C., et al. (2012) Exploring the transcriptome of ciliated cells using in silico dissection of human tissues. *PLoS One*, **7**, e35618.
2. Hoh, R. A., Stowe, T. R., Turk, E., et al. (2012) Transcriptional Program of Ciliated Epithelial Cells Reveals New Cilium and Centrosome Components and Links to Human Disease. *PLoS One*, **7**.
3. Ross, A. J., Dailey, L. A., Brighton, L. E., et al. (2007) Transcriptional profiling of mucociliary differentiation in human airway epithelial cells. *Am. J. Respir. Cell Mol. Biol.*, **37**, 169–85.
4. Rolfe, A. J., Bosco, D. B., Wang, J., et al. (2016) Bioinformatic analysis reveals the expression of unique transcriptomic signatures in Zika virus infected human neural stem cells. *Cell Biosci*., 6, **42**.
5. Zhang, F., Hammack, C., Ogden, S. C., et al. (2016) Molecular signatures associated with ZIKV exposure in human cortical neural progenitors. *Nucleic Acids Res*., **44**, 8610–8620.
6. Lima, M. C., de Mendonça, L. R., Rezende, A. M., et al. (2019) The transcriptional and protein profile from human infected neuroprogenitor cells is strongly correlated to zika virus microcephaly cytokines phenotype evidencing a persistent inflammation in the CNS. *Front. Immunol*., **10**.
7. Hong, E. P., Go, M. J., Kim, H. L., et al. (2017) Risk prediction of pulmonary tuberculosis using genetic and conventional risk factors in adult Korean population. *PLoS One*, **12**.
8. Myers, R. A., Scott, N. M., Gauderman, W. J., et al. (2014) Genome-wide interaction studies reveal sex-specific asthma risk alleles. *Hum. Mol. Genet*., **23**, 5251–5259.
9. Ibañez-Tallon, I., Heintz, N. and Omran, H. (2003) To beat or not to beat: Roles of cilia in development and disease. To beat or not to beat: Roles of cilia in development and disease. *Hum. Mol. Genet*. (2003) , **12**.
10. Torrelles, J. B. and Schlesinger, L. S. (2017) Integrating Lung Physiology, Immunology, and Tuberculosis. Integrating Lung Physiology, Immunology, and Tuberculosis. *Trends Microbiol.* (2017) , **25**, 688–697.
11. Tilley, A. E., Walters, M. S., Shaykhiev, R., et al. (2015) Cilia dysfunction in lung disease. *Annu. Rev. Physiol.*, **77**, 379–406.
12. Merchant, S. S., Prochnik, S. E., Vallon, O., et al. (2007) The Chlamydomonas genome reveals the evolution of key animal and plant functions. *Science* (**80**-.)., 318, 245–251.

13. Choksi, S. P., Lauter, G., Swoboda, P., et al. (2014) Switching on cilia: Transcriptional networks regulating ciliogenesis. Switching on cilia: Transcriptional networks regulating ciliogenesis. *Dev.* (2014) , **141**, 1427–1441.
14. Gros, J., Feistel, K., Viebahn, C., et al. (2009) Cell movements at Hensen's Node establish left/right asymmetric gene expression in the chick. *Science* (80-.)., 324, 941–944.
15. Ganapathiraju, M. K., Karunakaran, K. B. and Correa-Menéndez, J. (2016) Predicted protein interactions of IFITMs may shed light on mechanisms of Zika virus-induced microcephaly and host invasion. F1000Research, 5, 1919.
16. Bray, J. D., Chennathukuzhi, V. M. and Hecht, N. B. (2002) Identification and characterization of cDNAs encoding four novel proteins that interact with translin associated factor-X. *Genomics*, **79**, 799–808.

UNIPROTKB ACCESSION: Q9BPX7
GENE/PROTEIN NAMES: C7ORF25, UPF0415 PROTEIN

CONSERVATION

Eukaryota

•Metazoa

•Fungi: Pezizomycotina (Mostly Leotiomycota and Pezizomycetes, Tuber magnatum). Absent in *S. cerevisiae* and *Schizosaccharomyces pombe*

•Fungi incertae sedis (Mucoromycota (*Rhizopus microsporus*) and Chytridiomycota)

•Choanoflagellata, Amoebozoa (*Arcella intermedia*)

•Viridiplantae

•Excavata (Trypanosoma brucei)

Others: Cyanobacteria (K9QMI2) and Mimiviridae (M1PC36).

MODEL ORGANISMS:

M. musculus	AW209491/Q91WD4
G. gallus	A0A1D5P7R3
X. tropicalis	*TGas015c11*/Q5BKL1
X. laevis	*XB-GENE-5848830*/Q08AW5
D. rerio	zgc:55781/Q803H0
D. melanogaster	CG42553/Q9W0M6
C. elegans	F33G12.3/Q19987
A. thaliana	AT1G73380/A0A654EZB8

DOMAINS AND PTMS (HUMAN AND ORTHOLOGS)

- **UniprotKB, DUF1308** : PIN-like domain, structurally related to prokaryotic endoribonuclease VapC toxins (1). VapC proteins contain 4-9 acidic catalytic residues. C7orf25 contains less than 3 acidic residues, raising questions about the activity of the domain.
- **UniprotKB, DUF5614:** distantly related to PD-(D/E)XK nucleases, a superfamily involved in nucleic acid metabolism pathways like DNA degradation, recombination, repair and RNA processing (2). Nuclease activity not experimentally confirmed.
- **Human protein phosphorylated on Ser208, Ser210, Ser212 and Thr417** (3-7). Sites are conserved in mouse, but not in all the other eukaryotic orthologs, suggesting that they are dispensable for function.

SUBCELLULAR LOCATION (HUMAN AND MODEL ORGANISMS)

- **neXtProt and UniProtKB:** No annotation.
- **WoLF PSORT and DeepLoc1.0:** cytoplasm and/or nucleus. C7orf25 may shuttle between the nucleus and the cytosol to perform its activity.
- **NLS and NES:** no significant predictions. Nuclear proteins do not always have NLS/NES (8). C7orf25 could be transported in complexes with other proteins.

INTERACTIONS (HUMAN)

neXtProt: TARS1 and GTPBP1 in three yeast two hybrid experiments (9,10).

TARS1 (Threonine--tRNA ligase 1): catalyzes the attachment of threonine to tRNA(Thr) and edits incorrectly charged tRNA(Thr) at the post-transfer stage. Involved in: Trichothiodystrophy 7, non-photosensitive (TTD7). Subcellular location: Cytoplasm
GTPBP1 (GTP-binding protein 1): promotes the degradation of target mRNA species and plays a role in the regulation of circadian mRNA stability. May play a role in translational elongation by delaying aminoacyl tRNA accommodation in the ribosome A site and enabling the recruitment of the exosome (11). Subcellular location: Cytoplasm, Cytoplasmic exosome.

EXPRESSION (HUMAN AND MOUSE)

neXtProt:Validated at protein level: many peptides detected by mass spectrometry in a variety of tissues

HPA

No high quality data at the protein level
HPA RNA seq: low tissue specificity.

GENEVESTIGATOR:

Human microarray: High and medium in a wide range of tissues. Highest in gestational structures, nervous system, hematopoietic and immune cells.
Human RNAseq: High and medium in a wide range of tissues. Highest in nervous, circulatory, hematopoietic and immune, respiratory system.
Mouse microarray: High and medium in a wide range of tissues/systems. Highest in bone marrow hematopoietic cells, glial cells, brain neurons and spinal cord neurons.
Mouse RNAseq: High and medium in a wide range of tissues/systems. Highest in the nervous system, gestational structures, respiratory system.

COEXPRESSION (HUMAN)

GENEVESTIGATOR

RNA transcription and mRNA metabolic process, mitotic cell cycle, signal transduction and immune response, ubiquitin-proteasome system (UPS). In addition, regulation of cellular amino acid metabolic process, protein localization to vacuole, regulation of establishment of protein localization to mitochondria, regulation of cellular response to heat, viral process

SEEK

no significant GO term enrichment found.

PHENOTYPES/DISEASES (HUMAN AND ORTHOLOGS)

WormBase: reduction of fat content; lipid metabolism abnormal (WBRNAi00023454, RNAi). Caution: targets mainly adjacent gene F33G12.2.

PREDICTED FUNCTIONS

Based on the presence of the nuclease domains, the results of coexpression analysis and the interactions, we propose that C7orf25 is an **RNA binding protein** possessing **ribonuclease activity.** Coexpression analysis suggests a particular role in **DNA dependent transcription** and **mRNA metabolism.** Interaction with GTBP1 also suggests a role in **mRNA metabolism.** Based on both GTPBP1 and TARS1 interactions, one could also suggest a role in **tRNA metabolism** and **translation.** Expression analysis in human and mouse indicate expression in a wide range of tissues with highest levels in nervous and hematopoietic/immune system cells. Interestingly, mRNA and tRNA metabolic processes are known to play a critical role in these cells (12-13).

ADDITIONAL COMMENTS

In addition to RNA metabolism, the coexpression analysis showed a potential involvement in other biological functions but we believe this is due to the strong interplay between RNA metabolism and the UPS (14). Indeed, RNA binding proteins are involved in the UPS and the UPS has been shown to regulate complex RNA-dependent processes like mRNA processing and stability, ribosome associated quality control, tRNA metabolism as well as non-coding RNA features. Components of the UPS are known to play a critical role in the regulation of the cell cycle, signal transduction and immune response (15). As expected, the proteins annotated with mitotic cell cycle, signal transduction and immune response GO terms that coexpress with C7orf25 were all related to the UPS system.
The significance of the C7orf25 conservation profile in the frame of RNA metabolism hypothesis is difficult to evaluate, as PIN domains have been described as having enigmatic evolutionary profiles (16). C7orf25 is present in Chytridiomycota and Mucoromycota, representing basal lineages of fungi. Few genomic studies exist for these two phyla and most have focused on pathogenicity, making it difficult to assess the significance of the presence of C7orf25 in these phyla with respect to RNA metabolism. Mucoromycota are known to share a higher number of ancestral genes with metazoan genomes than with modern yeasts (Dikarya). Among Dikarya, C7orf25 is present in Ascomycota, but only in the subphylum Pezizomycotina and not in Saccharomycotina *(S. cerevisiae)* and Taphrinomycotina *(S. pombe)*. We did not find any association of this conservation profile in fungi with some specificity in RNA metabolism. In bacteria, PIN domain-containing proteins are usually involved in the toxin-antitoxin defense system (TA). The restricted presence of the DUF1308 domain in Cyanobacteria might reflect some specific TA system in this family. Finally, similar sequences were found in *Mimiviridae*. This family of viruses possesses a very large genome containing up to 1000 protein coding genes, many of them related to DNA repair and translation machinery, as well as chaperones related to DNA processing. Interestingly, *Mimiviridae* are the only known viruses encoding aminoacyl tRNA synthetases (17).

REFERENCES

1. Matelska, D., Steczkiewicz, K. and Ginalski, K. (2017) Comprehensive classification of the PIN domain-like superfamily. Comprehensive classification of the PIN domain-like superfamily. *Nucleic Acids Res.* **(2017)** , *45*, 6995–7020.
2. Laganeckas, M., Margeleviius, M. and Venclovas, E. (2011) Identification of new homologs of PD-(D/E)XK nucleases by support vector machines trained on data derived from profile-profile alignments. *Nucleic Acids Res.*, **39**, 1187–1196.
3. Wilhelm, M., Schlegl, J., Hahne, H., et al. (2014) Mass-spectrometry-based draft of the human proteome. *Nature*, **509**, 582–7.
4. Koboldt, D. C., Fulton, R. S., McLellan, M. D., et al. (2012) Comprehensive molecular portraits of human breast tumours. *Nature*, **490**, 61–70.
5. Liu, Z., Wang, F., Chen, J., et al. (2016) Modulating the selectivity of affinity absorbents to multi-phosphopeptides by a competitive substitution strategy. *J. Chromatogr. A*, **1461**, 35–41.
6. Phanstiel, D. H., Brumbaugh, J., Wenger, C. D., et al. (2011) Proteomic and phosphoproteomic comparison of human ES and iPS cells. *Nat. Methods*, **8**, 821–827.
7. Shiromizu, T., Adachi, J., Watanabe, S., et al. (2013) Identification of missing proteins in the neXtProt database and unregistered phosphopeptides in the phosphositeplus database as part of the chromosome-centric human proteome project. *J. Proteome Res.*, **12**, 2414–2421.
8. Freitas, N. and Cunha, C. (2009) Mechanisms and Signals for the Nuclear Import of Proteins. *Curr. Genomics*, **10**, 550–557.
9. Rolland, T., Taşan, M., Charloteaux, B., et al. (2014) A proteome-scale map of the human interactome network. *Cell*, **159**, 1212–1226.
10. Luck, K., Kim, D. K., Lambourne, L., et al. (2020) A reference map of the human binary protein interactome. *Nature*, **580**, 402–408.
11. Zinoviev, A., Goyal, A., Jindal, S., et al. (2018) Functions of unconventional mammalian translational GTPases GTPBP1 and GTPBP2. *Genes Dev.*, **32**, 1226–1241.
12. Nussbacher, J. K., Tabet, R., Yeo, G. W., et al. (2019) Disruption of RNA Metabolism in Neurological Diseases and Emerging Therapeutic Interventions. Disruption of RNA Metabolism in Neurological Diseases and Emerging Therapeutic Interventions. *Neuron* **(2019)** , *102*, 294–320.
13. Schott, J. and Stoecklin, G. (2010) Networks controlling mRNA decay in the immune system. Networks controlling mRNA decay in the immune system. *Wiley Interdiscip. Rev. RNA* **(2010)** , *1*, 432–456.
14. Thapa, P., Shanmugam, N. and Pokrzywa, W. (2020) Ubiquitin Signaling Regulates RNA Biogenesis, Processing, and Metabolism. Ubiquitin Signaling Regulates RNA Biogenesis, Processing, and Metabolism. *BioEssays* **(2020)** , *42*.
15. Nandi, D., Tahiliani, P., Kumar, A., et al. (2006) The ubiquitin-proteasome system. The ubiquitin-proteasome system. *J. Biosci.* **(2006)** , *31*, 137–155.
16. Anantharaman, V., Koonin, E. V. and Aravind, L. (2002) Comparative genomics and evolution of proteins involved in RNA metabolism. Comparative genomics and evolution of proteins involved in RNA metabolism. *Nucleic Acids Res.* **(2002)** , *30*, 1427–1464.
17. Abrahão, J. S., Araújo, R., Colson, P., et al. (2017) The analysis of translation-related gene set boosts debates around origin and evolution of mimiviruses. The analysis of translation-related gene set boosts debates around origin and evolution of mimiviruses. *PLoS Genet.* **(2017)** , *13*.

UNIPROTKB ACCESSION: Q96LI9
GENE NAMES: CXORF58, FLJ25444

CONSERVATION

•**Paralog: MFI (MFF interactor)**. Human MFI and CXorf58 have 14.6 % identity (38% in their N-terminal region)**.**

•**MFI:**

•Metazoa (including Porifera, Placozoa, Echinodermata, Mollusca, Annelida, Platyhelminthes, Hemichordata and Chordata) except Nematoda, Arthropoda and Cnidaria.

•Chlamydomonada (*Chlamydomonas reinhardtii* and Volvox)

•Ciliophora (*Paramecium tetraurelia* and *Tetrahymena thermophila)*

• Metamonada

•Fungi incertae sedis: Chytridiomycota (*Spizellomyces punctatus*).

•**CXorf58:**

•Metazoa (including Porifera, Placozoa, Cnidaria, Echinodermata, Mollusca, Annelida, Platyhelminthes, Hemichordata and Chordata) except Nematoda and Arthropoda

•Choanoflagellata (*Monosiga brevicollis* and *Salpingoeca rosetta*).

•Fungi incertae sedis: Chytridiomycota (*Spizellomyces punctatus*)

MODEL ORGANISMS :

M. musculus	AL646049/A0A5F8MPE6
G. gallus	Absent<br>Found in other birds such as *Nothoprocta perdicaria* (partridge)
D. rerio	gb:ee300746/XP_021332053 Dubious sequence that should be reexamined
X. tropicalis	XB-GENE-5614418/F6YKV3
D. rerio	gb:ee300746/XP_021332053

DOMAINS AND PTMS (HUMAN AND ORTHOLOGS)

HHPRED on human CXorf58 and MFI: similarity with the IQ motif of several proteins, a binding site for calmodulin and other EF-hand proteins in which the minimal core is (F,I,L,V)Qxxx(R,K) (1). The pattern [(F,I,L,V)Qxxx(R,K)] is indeed present in human MFI and all its orthologs. It is present in fish, Xenopus and fungal orthologs of CXorf58, but absent in all other CXorf58 orthologs including human (Q replaced by E/T and/or K/R replaced by L/Y/C). This is consistent with the fact that human MFI, but not CXorf58, was reported to interact with calmodulin in large scale two-hybrid studies (2).

SUBCELLULAR LOCATION (HUMAN AND MODEL ORGANISMS)

LITERATURE:

Human CXorf58: lower part of the head and midpiece of spermatids (3).
Mouse MFI: outer mitochondrial membrane (partial), cytosol (predominant) (4)
neXtProt and UniProtKB: No annotation for human CXorf58 and orthologs.

- **WoLF PSORT, DeepLoc1.0 and MitoProt II:** predictions too diverse to permit a clear conclusion.
- **TargetP2, PSORT II:** Predicted mitochondrial transit peptide for human CXorf58.
- **Predicted NLS** in different regions for human and model organisms. No consensus among the sequences.
- **No NES predicted**

INTERACTIONS (HUMAN)

neXtProt: None of high quality

EXPRESSION (HUMAN AND MOUSE)

neXtProt
Validation at protein level pending: only one peptide detected by mass spectrometry in sperm.

HPA

No high quality expression for CXorf58 and MFI at the protein level.
RNAseq: CXorf58 and MFI enriched in testis

GENEVESTIGATOR

- Human CXorf58 microarray: Low and medium in a wide range of systems. Highest in nose, testis and bronchoalveolar cells.
- Human CXorf58 RNAseq: High in testis, lung cells, hematopoietic and immune cells. Medium in a wide range of tissues.
- Highest in colonic microvessel endothelium.
- mouse CXorf58 ortholog. no data on ENSMUST00000239046

COEXPRESSION (HUMAN)

GENEVESTIGATOR

axoneme assembly, cilium movement, homophilic cell adhesion via plasma membrane adhesion molecules, microtubule based transport

SEEK

flagellated sperm motility, single cell fertilization and spermatogenesis

PHENOTYPES/DISEASES (HUMAN AND ORTHOLOGS)

None

PREDICTED FUNCTIONS

CXorf58 and MFI are paralogs. MFI was identified by comparative genomics as being present only in organisms having motile cilia (5). We found additional evidence suggesting a function of MFI in cilia. Mouse MFI expression is enriched in testis compared to other tissues and induced in meiotic spermatocytes and postmeiotic spermatids and tubules suggesting a role in male meiosis and gametogenesis (6). *D. rerio* MFI ortholog (C24H11orf65) is upregulated by foxj1, a transcription factor that has an evolutionarily conserved role as the master regulator of motile cilia biogenesis, and its inactivation leads to at least two motile cilia dysfunction-associated phenotypes (7, ZFIN). The MFI ortholog in *Paramecium* was detected in the cilium proteome (8). Human MFI is co-regulated with ciliary genes in primary ciliary dyskinesia patients (9). The presence of the IQ motif and the reported interactions with calmodulin suggest that MFI could regulate dynein motility on microtubules of cilia axonemes (10).
As for MFI, the absence of CXorf58 orthologs in Nematoda and Arthropoda, that have lost the ability to grow motile cilia indicates that CXorf58 may be involved in **motile cilia assembly or function.** Coexpression analysis supports this hypothesis. SEEK coexpression analysis also indicates a possible involvement in sexual reproduction and more particularly in **spermatogenesis**. This result can be related to the potential role of the protein in cilia since flagellar assembly and movement are known to be important for both sperm maturation and motility. Tissue expression data partially supports these hypotheses as the highest expression was observed in tissues containing motile cilia like testis, lung and nose cells. However, Genevestigator data also suggests the presence of CXorf58 in colonic microvessel endothelium and myeloïde leukocyte cells that do not contain motile cilia. Taken together, our observations suggest that both MFI and CXorf58 would be involved in motile cilia-related functions. Cilia are subject to an important variability within and between eukaryotic phyla in terms of number, length, position on the cell surface, structural and molecular composition, but also in terms of calcium regulation (11). The loss of the IQ motif in most CXorf58 proteins may be correlated to such evolutionary change in calcium regulation.

ADDITIONAL COMMENTS

MFI was shown to partially localize to the outer mitochondrial membrane and to play a role as an inhibitor of mitochondrial fission (4). Calcium and dynein are important players of mitochondria dynamics (11). The presence of a calmodulin binding site (IQ motif) in MFI could therefore explain its dual function in both cilia motility and mitochondria.
Although human CXorf58 was found at the lower part of the head and midpiece of spermatids, which is compatible with an association of CXorf58 to mitochondria, subcellular predictions are too diverse to permit a clear conclusion and should be confirmed experimentally before assessing a potential role of CXorf58 in mitochondrial function.
In UniProtKB entry A0A5F8MPE6, the mouse ortholog of CXorf58 has been wrongly named Fam90a1b instead of AL646049. In MGI, Fam90a1b is wrongly assigned as CXorf58 ortholog. Fam90a1b and AL646049 are overlapping genes. Fam90a1b is part of the multi-copy gene family FAM90. The majority of the human genes from this family are annotated in UniProtKB as potential pseudogenes.

REFERENCES

1. Rhoads, A. R. and Friedberg, F. (1997) Sequence motifs for calmodulin recognition. *FASEB J.*, **11**, 331–340.
2. Luck, K., Kim, D. K., Lambourne, L., et al. (2020) A reference map of the human binary protein interactome. *Nature*, **580**, 402–408.
3. Vandenbrouck, Y., Lane, L., Carapito, C., et al. (2016) Looking for Missing Proteins in the Proteome of Human Spermatozoa: An Update. *J. Proteome Res.*, **15**, 3998–4019.
4. Lee, J., Pappalardo, Z., Chopra, D. G., et al. (2018) A genetic interaction map of insulin production identifies Mfi as an inhibitor of mitochondrial fission. *Endocrinology*, **159**, 3321–3330.
5. Merchant, S. S., Prochnik, S. E., Vallon, O., et al. (2007) The Chlamydomonas genome reveals the evolution of key animal and plant functions. *Science (80-.).*, **318**, 245–251.
6. Chalmel, F., Rolland, A. D., Niederhauser-Wiederkehr, C., et al. (2007) The conserved transcriptome in human and rodent male gametogenesis. *Proc. Natl. Acad. Sci. U. S. A.*, **104**, 8346–8351.
7. Choksi, S. P., Babu, D., Lau, D., et al. (2014) Systematic discovery of novel ciliary genes through functional genomics in the zebrafish. *Dev.*, **141**, 3410–3419.
8. Arnaiz, O., Malinowska, A., Klotz, C., et al. (2009) Cildb: a knowledgebase for centrosomes and cilia. *Database (Oxford).*, **2009**, bap022.
9. Geremek, M., Bruinenberg, M., Ziętkiewicz, E., et al. (2011) Gene expression studies in cells from primary ciliary dyskinesia patients identify 208 potential ciliary genes. *Hum. Genet.*, **129**, 283–93.
10. Inaba, K. (2015) Calcium sensors of ciliary outer arm dynein: functions and phylogenetic considerations for eukaryotic evolution. *Cilia*, **4**, 6.
11. Szabadkai, G., Simoni, A. M., Bianchi, K., et al. (2006) Mitochondrial dynamics and Ca2+ signaling. Mitochondrial dynamics and Ca2+ signaling. *Biochim. Biophys. Acta - Mol. Cell Res.* **(2006)** , *1763*, 442–449.

UNIPROTKB ACCESSION: Q9BUV0
GENE/PROTEIN NAMES: RSRP1, ARGININE/SERINE-RICH PROTEIN 1, C1ORF63

CONSERVATION

Gnathostomata (Jawed vertebrates).

MODEL ORGANISMS:

M. musculus	2700043I21Rik/D4Ucla2/D4Wsu53e/ Q3UC65
G. gallus	A0A3Q2U4I5
D. rerio	wu:fc15g08/zgc:136474/Q1ECZ9

DOMAINS AND PTMS (HUMAN AND ORTHOLOGS)

- Rich in arginine and serine residues.
- Phosphorylated at Ser274 and Ser282 in breast tumor tissues (1) and at Ser12 in HeLa cells (2).
- Methylated at Arg135 and ubiquitinated at Lys188. These sites are conserved in most of the orthologs.

SUBCELLULAR LOCATION (HUMAN AND MODEL ORGANISMS)

- **neXtProt and UniProtKB:** No annotation
- **WoLF PSORT and DeepLoc:** nuclear
- **SecNLS and NLStradamus:** one or two NLS. The NLS predicted by NLStradamus are long stretches of basic amino acid residues and dubious because of their size (3).
- **netNES but not LocNES** predicts a nuclear export signal (*H. sapiens* and *M. musculus).*

The putative NLS and NES should be experimentally confirmed.

INTERACTIONS (HUMAN)

CCNL1 (two hybrid, 4), **CLK2** (two hybrid, 5-6; protein complementation assay, 4) and **CLK3** (two hybrid, 4-7).
These nuclear proteins are involved in pre-mRNA splicing. CLK2 and CLK3 are serine/threonine/tyrosine kinases that phosphorylate spliceosomal serine- and arginine-rich proteins. CCNL1 is a cyclin that functions in association with cyclin-dependent kinases (CDKs) and plays a role in the regulation of RNA polymerase II (neXtProt overview).
CLK2, highly expressed in breast tumors was suggested to act as an oncogene (8)

EXPRESSION (HUMAN AND MOUSE)

LITERATURE

Highly expressed in breast tumors (9).
Upregulated after induction of cell cycle arrest in human T cells (10).

NEXTPROT

Validated at protein level: many peptides detected by mass spectrometry in a variety of tissues.

HPA

No high quality data at the protein level
RNAseq: Low tissue specificity.

GENEVESTIGATOR

High and wide in human and *M. musculus* (mouse) (RNAseq and microarrays)

COEXPRESSION (HUMAN)

GENEVESTIGATOR

RNA splicing, peptidyl-serine modification and positive regulation of transcription, DNA-templated. CLK2 is among the coexpressing genes.

SEEK

chromatin organization, RNA splicing and regulation of mRNA processing. CCNL1 is among the coexpressing genes.

PHENOTYPES/DISEASES (HUMAN AND ORTHOLOGS)

Glu239Gly (dbSNP=rs1043879): increased erythrocyte sedimentation rate (11).

PREDICTED FUNCTIONS

Based on the interactions with nuclear proteins involved in pre-mRNA splicing (CCNL1, CLK2 and CLK3), its induction upon cell cycle arrest and coexpression profiles, we propose that RSRP1 is involved in **mRNA splicing** and in the **regulation of cell cycle**. Based on coexpression data and one the functions described for CCNL1, we suggest that RSRP1 is involved in **regulation of transcription by RNA polymerase II**. The coexpression with proteins involved in chromatin organization is consistent with evidence suggesting that chromatin modifications may guide the spliceosome in the detection of exons among the intronic sequences (12). The function and/or localization of RSRP1 may be regulated by phosphorylation and methylation. Many arginine-methylated proteins have been found to interact with nucleic acids (13**).** This conclusion is in accordance with the predicted nuclear subcellular location.

ADDITIONAL COMMENTS

We reject the hypothesis that RSRP1 is phosphorylated by CLK2 or CLK3 at Ser12, Ser274 or Ser282. The substrate specificity for CLK2 and CLK3 indicates a moderate preference for hydrophobic amino acids at position P+1 (Phe being preferred but also Pro, Leu, and Val), a preference for hydrophobic (Ala, Leu, Ile, Phe, Val) or a polar (Gln, Gly, and Tyr) but not charged amino acid at position P-2 and Arg at position P-3 (14). While RSRP1 has Pro at P+1 for Ser12, Ser274 and Ser282, positions P-2 and P-3 do not have the required residues for CLK2 or CLK3 phosphorylation (Trp-ProGly-Ser12-Pro, SerSerArg-Ser274-Pro and LysLysLys-Ser282-Pro). The association of rs1043879 (Glu239Gly) with increased erythrocyte sedimentation rate, a commonly performed test of the acute phase response (11), is also difficult to explain with the current data. We can not explain the evolutionary reason for the absence in Xenopus either.

REFERENCES

1. Koboldt, D. C., Fulton, R. S., McLellan, M. D., et al. (2012) Comprehensive molecular portraits of human breast tumours. *Nature*, **490**, 61–70.
2. Sharma, K., D'Souza, R. C. J., Tyanova, S., et al. (2014) Ultradeep Human Phosphoproteome Reveals a Distinct Regulatory Nature of Tyr and Ser/Thr-Based Signaling. *Cell Rep.*, **8**, 1583–1594.
3. Nguyen Ba, A. N., Pogoutse, A., Provart, N., et al. (2009) NLStradamus: A simple Hidden Markov Model for nuclear localization signal prediction. *BMC Bioinformatics*, **10**.
4. Luck, K., Kim, D. K., Lambourne, L., et al. (2020) A reference map of the human binary protein interactome. *Nature*, **580**, 402–408.
5. Rolland, T., Taşan, M., Charloteaux, B., et al. (2014) A proteome-scale map of the human interactome network. *Cell*, **159**, 1212–1226.
6. Fragoza, R., Das, J., Wierbowski, S. D., et al. (2019) Extensive disruption of protein interactions by genetic variants across the allele frequency spectrum in human populations. *Nat. Commun.*, **10**.
7. Rual, J.-F., Venkatesan, K., Hao, T., et al. (2005) Towards a proteome-scale map of the human protein-protein interaction network. *Nature*, **437**, 1173–8.
8. Yoshida, T., Kim, J. H., Carver, K., et al. (2015) CLK2 is an oncogenic kinase and splicing regulator in breast cancer. *Cancer Res.*, **75**, 1516–1526.
9. Hong, C. Q., Zhang, F., You, Y. J., et al. (2015) Elevated C1orf63 expression is correlated with CDK10 and predicts better outcome for advanced breast cancers: A retrospective study. *BMC Cancer*, **15**.
10. Chechlinska, M., Siwicki, J. K., Gos, M., et al. (2009) Molecular signature of cell cycle exit induced in human T lymphoblasts by IL-2 withdrawal. *BMC Genomics*, **10**.
11. Kullo, I. J., Ding, K., Shameer, K., et al. (2011) Complement receptor 1 gene variants are associated with erythrocyte sedimentation rate. *Am. J. Hum. Genet.*, **89**, 131–138.
12. Gómez Acuña, L. I. and Kornblihtt, A. R. (2014) Long range chromatin organization: A new layer in splicing regulation? *Transcription*, **5**.
13. Wesche, J., Kühn, S., Kessler, B. M., et al. (2017) Protein arginine methylation: a prominent modification and its demethylation. Protein arginine methylation: a prominent modification and its demethylation. *Cell. Mol. Life Sci.* **(2017)** , *74*, 3305–3315.
14. Bullock, A. N., Das, S., Debreczeni, J. É., et al. (2009) Kinase Domain Insertions Define Distinct Roles of CLK Kinases in SR Protein Phosphorylation. *Structure*, **17**, 352–362.

UNIPROTKB ACCESSION: H3BR10
GENE/PROTEIN NAMES: SMLR1, SMALL LEUCINE-RICH PROTEIN 1

CONSERVATION

Paralog: ADIG (retrieved as reciprocal best hits by HHPRED but not by protein BLAST or psiBLAST). Identity: human SMLR1 and ADIG 32%; mouse SMLR1 and ADIG 24 %.
SMLR1 conserved in jawed vertebrates.

MODEL ORGANISMS:

M. musculus	2010003K15Rik/J3QMJ4
X. tropicalis	*XR_208637.4*
D. rerio	*XR_222668.4*
G. gallus	*XR_211919.3*

Annotated as non-coding RNA in *X. tropicalis*, *D. rerio* and *G. gallus* in RefSeq. The *X. laevis* genomic sequence is dubious and should be reexamined.
As there is evidence of mRNA expression in many vertebrate species (*Anas platyrhynchos* XM_021270352, *Lacerta agilis* XM_033145416, *Chelonoidis abingdonii* XM_032772684, *Rhinatrema bivittatum* XM_029594562, *Anguilla anguilla* XM_035422030, *Paramormyrops kingsleyae* XM_023797034)**,** the RefSeq annotations in *X. tropicalis*, *D. rerio* and *G. gallus* should be revised.
Possible initiation at Met38 rather than Met1: N-terminal region not conserved in orthologs, and no tryptic peptide identified by mass spectrometry in this region in the human protein (neXtProt).

DOMAINS AND PTMS (HUMAN AND ORTHOLOGS): -

SUBCELLULAR LOCATION (HUMAN AND MODEL ORGANISMS)

- **neXtProt:** Two transmembrane domains in the human protein. One of these transmembrane domains resides in the N-terminal region only common to primate sequences.
- **UniProtKB**: One transmembrane domain in *M.musculus*.
- **TMHMM and Phobius:** One transmembrane domain in *X. tropicalis*, *D. rerio* and *G. gallus*.
- **WoLF PSORT:** secreted, then plasma membrane (*H.sapiens* and *M.musculus)*.
- DeepLoc: endoplasmic reticulum (ER) membrane, then Golgi apparatus or secreted (*H.sapiens*, *M.musculus*, *X.tropicalis* and *G.gallus*).
- **SecretomeP-2.0:** no.
- **neXtProt:** no evidence for secretion in plasma or urine proteomics datasets.

No clear consensus among the localization prediction programs, the subcellular location of SMLR1 should be experimentally analysed.

INTERACTIONS (HUMAN)

BSCL2 (two hybrid, 1): localizes at ER-lipid droplets contact sites and is involved in formation of lipid droplets and in the differentiation and development of adipocytes (neXtProt). Mutations in BSCL2 result in lipodystrophy (CGL2, MIM:269700), spastic paraplegia (SPG17, MIM:270685), neuromuscular disease (HMN5A, MIM:600794) and encephalopathy (PELD, MIM:615924).
BSCL2 knockdown leads to increased triglyceride accumulation in the liver HepG2 cell line (2) and mice with BSCL2 gene deletion showed an increase in liver triglycerides. However, mice with a liver-specific deletion of the BSCL2 gene did not develop hepatic steatosis, indicating that BSCL2 is not autonomous to liver lipid homeostasis (3).

EXPRESSION (HUMAN AND MOUSE)

NEXTPROT

Validated at protein level: several peptides detected by mass spectrometry in a variety of tissues

HPA

No high quality data at the protein level (HPA).
HPA RNAseq: RNA tissue specificity: Group enriched (intestine, liver).

GENEVESTIGATOR

- **Human microarrays**: high in liver and small intestine. Medium in all the systems.
- **Human RNAseq**: high in liver, extrahepatic and intrahepatic bile duct, cerebral cortex microglia cell and hepatic flexure. Medium in small intestine, bronchoalveolar system cell, hippocampus astrocyte, Peyer's patch, cerebral cortex myelinating oligodendrocyte and kidney.
- **Mouse microarrays**: high in liver, small intestine, large intestine, kidney. Medium in most systems.
- **Mouse RNAseq**: high in liver, small intestine, kidney, brainstem microglia cell, patellar tendon cell. Medium in most systems.

COEXPRESSION (HUMAN)

GENEVESTIGATOR

lipid metabolism, transport, assembly, remodeling and homeostasis, other liver or kidney related metabolic processes, small molecule metabolic processes and other processes (oxidative demethylation, regulation of humoral immune response, transmembrane transport)

SEEK

no data

PHENOTYPES/DISEASES (HUMAN AND ORTHOLOGS)

Val62Met (dbSNP= rs1044303): decreased bone mineral density, a marker of osteoporosis (4)

PREDICTED FUNCTIONS

ADIG, the SMLR1 paralog, was suggested to regulate adipocyte differentiation and modulate the expression and secretion of leptin (LEP), an adipocyte-secreted hormone (5). This proposed function derives from studies performed on mouse or bovine models, and to our knowledge no studies were done in human. While mouse Adig expression is highest in adipose tissue, human ADIG is highly expressed in testes, suggesting an additional or different function in human. SMLR1 is not enriched in testis or adipose tissue but in liver and intestine both in human and mouse, suggesting that ADIG and SMLR1 may not be functionally redundant. Human SMLR1 is coregulated with proteins involved in lipid metabolism, suggesting that it may be involved in **lipid metabolic or homeostasis processes** in the liver and intestine**.** The localization to the ER membrane predicted by DeepLoc would support the interaction with BSCL2. However, the physiological relevance of this interaction should be analyzed in detail because the function of BSCL2 does not seem to be autonomous to the liver. The relevance of rs1044303 (Val62Met) for bone mineral density is worth investigating further because liver disease is one of the secondary causes of osteoporosis (6).

REFERENCES

1. Rolland, T., Taşan, M., Charloteaux, B., et al. (2014) A proteome-scale map of the human interactome network. *Cell*, **159**, 1212–1226.
2. Li, Q., Li, Y., Zhang, Z., et al. (2019) SEIPIN overexpression in the liver may alleviate hepatic steatosis by influencing the intracellular calcium level. *Mol. Cell. Endocrinol.*, **488**, 70–78.
3. McIlroy, G. D., Mitchell, S. E., Han, W., et al. (2020) Ablation of Bscl2/seipin in hepatocytes does not cause metabolic dysfunction in congenital generalised lipodystrophy. *DMM Dis. Model. Mech.*, *13*.
4. Morris, J. A., Kemp, J. P., Youlten, S. E., et al. (2019) An atlas of genetic influences on osteoporosis in humans and mice. *Nat. Genet.*, **51**, 258–266.
5. Kilpeläinen, T. O., Carli, J. F. M., Skowronski, A. A., et al. (2016) Genome-wide meta-analysis uncovers novel loci influencing circulating leptin levels. *Nat. Commun.*, **7**.
6. Jeong, H. M. and Kim, D. J. (2019) Bone diseases in patients with chronic liver disease. Bone diseases in patients with chronic liver disease. *Int. J. Mol. Sci.* (2019) , **20**.

UNIPROTKB ACCESSION: Q6P2H8
GENE/PROTEIN NAMES: TMEM53, NET4, NUCLEAR ENVELOPE TRANSMEMBRANE PROTEIN 4

CONSERVATION

Metazoa, Fungi (*Penicillium sp)*, Viridiplantae, Choanoflagellata and Amoebozoa.

MODEL ORGANISMS

M. musculus	1110038M16Rik/1500001P22Rik/1700012A05Rik/Q9D0Z3
G. gallus	A0A1L1RPU6
X. tropicalis	A9JSB6
X. laevis	paralogs: Q6DJC8 and Q5PPS7
D. rerio	zgc:92204/Q6DHN0
D. melanogaster	paralogs: CG8245/A1Z6G9 and lethal(2) k09913/A0A0B4LGC6
C. elegans	paralogs: C09B8.4/Q17846; CELE_T24H10.4/Q22750;Q9TZH8 and Q9TZH9
A. thaliana	paralogs: At2g18245/A0A178VZN4 and At3g19970/Q9LHE8
Dictyostelium discoideum	paralogs: Q54YR8/Q54N85 (BLAST results)
S. cerevisiae and *Schizosaccharomyces pombe*	absent

C. elegans Q9TZH9, *D. melanogaster* A0A0B4LGC6 and *A. thaliana* Q9LHE8, with weak BLAST scores and not considered as orthologs by OMA, were not analysed further.

DOMAINS AND PTMS (HUMAN AND ORTHOLOGS)

- **InterPro: IPR029058**, alpha/beta hydrolase fold, found in hydrolytic enzymes and in “moonlighting” proteins that are able to perform different functions by interacting with different partners (1).
- **UniProtKB: DUF829 family**, evolutionary close to Family of serine hydrolase 1 (FSH1) (ESTHER database, 2). Human member of FSH1: OVCA2, esterase with strong preference for unbranched alkyl ester substrates greater than 10 carbons (3).
- **HHPRED and SWISS-MODEL:** similarity with lipases and esterases. Predicted catalytic residues: Ser113, Asp220 and His252 (conserved in orthologs).
- **SWISS-MODEL template:** putative lipase from *Acinetobacter baumannii* (4OPM.1.A/B0V9K7). This lipase crystallizes with polyethylene glycol inside its structure. Part of the residues that are analogous to the annotated transmembrane domain in TMEM53 make contact with the polymer, suggesting that part of the annotated transmembrane domain may form a binding pocket for lipids or other alkyl esters.

SUBCELLULAR LOCATION (HUMAN AND MODEL ORGANISMS)

LITERATURE

Associated with outer nuclear membrane in human cell lines and in rat liver, but not integral to membrane (4). Moves from the ER and outer nuclear membrane to lipid droplets upon induction with fatty acids in mammalian cell lines and in *D. discoideum* (Q54YR8/DDB0204431, 5).
TMEM53 was predicted and experimentally shown to be mitochondrial in *C.elegan*s (Q9TZH8, 6).

- **neXtProt and UniProtKB:** human and vertebrate orthologs annotated with a transmembrane domain located in the alpha/beta hydrolase fold. The alpha/beta hydrolase fold is intrinsically soluble and proteins with the alpha/beta hydrolase fold that interact with membranes generally do so with additional N- or C-terminal domains (1). This region may not be a transmembrane domain, because it would break the folding. In addition, experimental findings show that the protein is not integral to membranes. The association with membranes may occur by hydrophobic interactions or by interaction with an unidentified protein because no evidence for attachment to the membrane by GPI-anchor could be found for *H. sapiens* and any of the orthologs.
- **WoLF PSORT:** plasma membrane (*H. sapiens* and most orthologs).
- **DeepLoc:** peroxisomal membrane (all vertebrates except *G. gallus* predicted at the mitochondrial membrane) and then ER membrane (mammalian and Xenopus). Chloroplast membrane for *A. thaliana*. Unreliable prediction to the

mitochondria for *G. gallus* due to lack of conservation in the rest of the vertebrate orthologs.

INTERACTIONS (HUMAN)

neXtProt: None of high quality

EXPRESSION (HUMAN AND MOUSE)

neXtProt
Validated at protein level: several peptides detected by mass spectrometry in a variety of tissues

HPA

No high quality data at the protein level
RNAseq: Tissue enhanced (blood, liver).

GENEVESTIGATOR

- **Human microarrays**: medium or high in all systems. Highest in hematopoietic cells, kidney, intestine, liver, tarsal conjunctiva, respiratory epitheliums, urinary bladder urothelium, dermal lymphatic microvessel endothelial cell, cell growth plate, fetal chondrocyte, foreskin-derived neonatal fibroblast, prostatic stroma cell, right ventricle interventricular septum and oocyte.
- **Human RNAseq**: high in all systems. Highest in duodenum, testis, hepatic flexure, transverse colonic mucosa. Medium in all systems.
- Mouse microarrays: high in all systems. Highest in testis, spermatid, aortic arch, liver, horizontal basal neural stem cell, heart left ventricle, nasal epithelium, tracheal epithelium cell, embryonic soft anterior palate, tracheal basal cell, adrenal gland, adipose tissue.
- **Mouse RNAseq**: high in all systems. Highest in testis, subcutaneous lymph node fibroblastic reticular cell, cortical thymic epithelium cell, gonadal adipose tissue, lumbar dorsal root ganglion neuron, adrenal gland, bone marrow stromal cell, white adipose tissue, liver, mesenteric adipose tissue macrophage.

COEXPRESSION (HUMAN)

GENEVESTIGATOR

lipid metabolism, transport, absorption and homeostasis, carbohydrate-related transport, xenobiotic metabolic process, alcohol metabolic process and oxidation-reduction process

SEEK

lipid metabolism, transport, absorption and homeostasis, acetyl-CoA and tricarboxylic acid cycle-related processes, carbohydrate-related processes, wound healing and coagulation, inflammation and immune response, heme catabolic process, drug and xenobiotic metabolic process, aminoacid metabolism, peroxisome organization.

The following processes with lower fold enrichment have not been considered further: regulation of endopeptidase activity, hormone metabolism, organophosphate ester transport, liver development, intracellular receptor signaling pathway.

The following very broad terms were also considered as not relevant: secondary metabolic process, monocarboxylic acid catabolic process, organic hydroxy compound catabolic process.

PHENOTYPES/DISEASES (HUMAN AND ORTHOLOGS)

- Knockdown of TMEM53 by RNAi in MRC-5 fetal lung fibroblasts and the U-2 OS cells resulted in cell cycle withdrawal (7).
- Recessive lethal in *D. melanogaste*r for k09913.
- No phenotype in reproduction, morphology and lethality tests in *C.elegans* (RNAi, Q22750 and Q17846)
- Myosin organization defects in the wall muscle in *C.elegans* (RNAi, Q9TZH8, 8).
- Together with the mitochondrial localization of Q9TZH8, the myosin organization defect observed upon Q9TZH8 downregulation could be the result of specific functional divergence among the worm paralogs. This is supported by the observation that muscle is not among the tissues with highest expression in humans.
- All rice paralogs are induced in various stress conditions (9), but the relevance of these findings for the human

protein is limited. Interestingly, proteomics studies show that lipid droplets in Arabidopsis contain proteins involved in plant response to biotic stress (10).

PREDICTED FUNCTIONS

Based on the predicted alpha/beta hydrolase fold, its homology with lipases, its localization in lipid droplets upon fatty acids induction in *Dictyostelium* and human cell lines and its coexpression with proteins involved in lipid metabolism, we suggest that TMEM53 is a **lipase** involved in **lipid metabolic processes**. TMEM53 may exert its function in association with lipid droplets in contact with the ER and/or the peroxisome. Coexpression with genes coding for proteins involved in peroxisome organization and the subcellular location prediction by DeepLoc to peroxisomes are interesting because the peroxisomes are tightly linked to lipid droplets (11). The subcellular location prediction to the chloroplast for the Arabidopsis ortholog is compatible with our hypothesis because the chloroplast is one of the sites for the synthesis of lipids. In addition, in plants, lipid droplets are found in seeds and leaves, both in contact with the ER and the chloroplast (10). The cycle withdrawal phenotype observed in human cell lines after RNAi could be the consequence of disturbing lipid metabolism. Indeed, energetic status and metabolism are known to be critical parameters for the cell's decision to enter mitosis (12). Carbohydrates and lipids are a source of energy and it is not surprising that proteins involved in carbohydrate related processes are among the genes coexpressed with TMEM53. Heme containing proteins such as cytochrome P450 proteins are involved in lipid and bile acid metabolism (13-14) explaining the coexpression with proteins involved in heme metabolism. The coexpression with proteins involved in wound healing, coagulation, inflammation and immune response could be explained by the influence of plasma lipids and lipoproteins on both procoagulant and anticoagulant reactions in plasma (15). Overall, the coexpression profile indicates that the expression of TMEM53 seems tightly regulated by the metabolic state of the cell.

ADDITIONAL COMMENTS

The reason why testis is the tissue with the highest expression of TMEM53 in mouse but not in human is elusive for us, but we speculate it may reflect a different metabolic regulation in mouse testis.
Even if most of the gathered information converge towards a role of TMEM53 as a lipase involved in lipid metabolism or homeostasis, one cannot exclude that TMEM53 might be an esterase with substrates other than lipids, as TMEM53 structure can be modeled with esterases other than lipases.
Some of the TMEM53 homologs found in *Penicillium sp*. are annotated as indole-diterpene biosynthesis protein PaxU because in *Penicillium paxilli,* the gene (called PP112) localizes in the PAX locus. The PAX locus contains 22 genes of which 8 may have a role in the indole-diterpene paxilline biosynthesis, but PP112 is probably not involved in that pathway (16). In *Penicillium soppi*, the ortholog of TMEM53 belongs to a biosynthetic gene cluster that contains 4 other genes, 3 of them sufficient to produce alkylresorcinols (soppiline B and C) and one polyketide (soppiline A) (17), but the putative function of the TMEM53 ortholog in this biosynthetic pathway was not assessed.
Loss-of-function variants in TMEM53 were recently identified in four independent families with sclerosing bone disorder (18). We did not predict a role in skeletal function for TMEM53, but there is evidence that intracellular lipid droplets and lipid metabolism affect osteoblast function (19). It would be interesting to know if other functions linked to lipid metabolism are affected in these patients. The BMP-SMAD signaling pathway was found to be dysregulated in TMEM53 knock-out human cell lines and mutant mice, and authors suggested that TMEM53 may act by blocking the cytoplasm-nucleus translocation of BMP2-activated SMAD proteins (18). Although BMP signaling pathway is involved in adipogenesis (20), nothing in our data mining led us to predict a function of TMEM53 in SMAD-BMP signaling pathway or cytoplasm-nucleus protein translocation. In particular, the conservation of TMEM53 in fungi and plants suggests that its functions are not restricted to BMP signaling pathway (GO:0030509) or SMAD protein signal transduction (GO:0060395), which are only found in metazoa.

REFERENCES

1. Marchot, P. and Chatonnet, A. (2012) Enzymatic Activity and Protein Interactions in Alpha/Beta Hydrolase Fold Proteins: Moonlighting Versus Promiscuity. *Protein Pept. Lett.*, **19**, 132–143.
2. Lenfant, N., Hotelier, T., Velluet, E., et al. (2013) ESTHER, the database of the α/β-hydrolase fold superfamily of proteins: Tools to explore diversity of functions. *Nucleic Acids Res.*, **41**.
3. Bun, J. S., Slack, M. D., Schemenauer, D. E., et al. (2020) Comparative analysis of the human serine hydrolase OVCA2 to the model serine hydrolase homolog FSH1 from S. Cerevisiae. *PLoS One*, **15**.
4. Malik, P., Korfali, N., Srsen, V., et al. (2010) Cell-specific and lamin-dependent targeting of novel transmembrane proteins in the nuclear envelope. *Cell. Mol. Life Sci.*, **67**, 1353–1369.
5. Du, X., Barisch, C., Paschke, P., et al. (2013) Dictyostelium lipid droplets host novel proteins. *Eukaryot. Cell*, **12**, 1517–1529.
6. Meissner, B., Rogalski, T., Viveiros, R., et al. (2011) Determining the sub-cellular localization of proteins within caenorhabditis elegans body wall muscle. *PLoS One*, **6**.

7. Korfali, N., Srsen, V., Waterfall, M., et al. (2011) A flow cytometry-based screen of nuclear envelope transmembrane proteins identifies NET4/tmem53 as involved in stress-dependent cell cycle withdrawal. *PLoS One*, **6**.
8. Meissner, B., Warner, A., Wong, K., et al. (2009) An integrated strategy to study muscle development and myofilament structure in Caenorhabditis elegans. *PLoS Genet.*, **5**.
9. Li, L., Lv, M., Zhao, L., et al. (2018) Molecular characterization and function analysis of the rice OsDUF829 family. *Biotechnol. Biotechnol. Equip.*, **32**, 550–557.
10. Brocard, L., Immel, F., Coulon, D., et al. (2017) Proteomic Analysis of Lipid Droplets from Arabidopsis Aging Leaves Brings New Insight into Their Biogenesis and Functions. *Front. Plant Sci.*, **8**, 894.
11. Joshi, A. S. and Cohen, S. (2019) Lipid Droplet and Peroxisome Biogenesis: Do They Go Hand-in-Hand? *Front. cell Dev. Biol.*, **7**, 92.
12. Fajas, L. (2013) Re-thinking cell cycle regulators: the cross-talk with metabolism. *Front. Oncol.*, **3**, 4.
13. Neuschwander-Tetri, B. A. (2015) Retinoid X receptor: The forgotten partner in regulating lipid metabolism? Retinoid X receptor: The forgotten partner in regulating lipid metabolism? *Am. J. Clin. Nutr.* **(2015)** , *102*, 5–6.
14. Finn, R. D., Henderson, C. J., Scott, C. L., et al. (2009) Unsaturated fatty acid regulation of cytochrome P450 expression via a CAR-dependent pathway. *Biochem. J.*, **417**, 43–54.
15. Deguchi, H., Elias, D. J. and Griffin, J. H. (2017) Minor Plasma Lipids Modulate Clotting Factor Activities and May Affect Thrombosis Risk. *Res. Pract. Thromb. Haemost.*, **1**, 93–102.
16. Scott, B., Young, C. A., Saikia, S., et al. (2013) Deletion and gene expression analyses define the paxilline biosynthetic gene cluster in Penicillium paxilli. *Toxins (Basel).*, **5**, 1422–46.
17. Navarro-Muñoz, J. C. and Collemare, J. (2019) Evolutionary Histories of Type III Polyketide Synthases in Fungi. *Front. Microbiol.*, **10**, 3018.
18. Guo, L., Iida, A., Bhavani, G. S. L., et al. (2021) Deficiency of TMEM53 causes a previously unknown sclerosing bone disorder by dysregulation of BMP-SMAD signaling. *Nat. Commun.*, **12**.
19. Alekos, N. S., Moorer, M. C. and Riddle, R. C. (2020) Dual Effects of Lipid Metabolism on Osteoblast Function. *Front. Endocrinol. (Lausanne).*, **11**, 578194.
20. Blázquez-Medela, A. M., Jumabay, M. and Boström, K. I. (2019) Beyond the bone: Bone morphogenetic protein signaling in adipose tissue. Beyond the bone: Bone morphogenetic protein signaling in adipose tissue. *Obes. Rev.* **(2019)** , *20*, 648–658.

UNIPROTKB ACCESSION: C9JQI7
GENE/PROTEIN NAMES: TMEM232, FLJ54480

CONSERVATION

Metazoa, including Porifera, Cnidaria, Placozoa, Echinodermata, Mollusca, Annelida, Platyhelminthes, Hemichordata and Chordata. Absent in Aves, Arthropoda and Nematoda.

MODEL ORGANISMS:

M. musculus	E130009J12Rik/LOC381107/Q5K6N0
X. laevis	A0A1L8I1Q0
X. tropicalis	A0A6I8S0J9/F6SF94 sequences are fragments
D. rerio	si:dkey-264m17.1/A5WVS1

DOMAINS AND PTMS (HUMAN AND ORTHOLOGS): -

SUBCELLULAR LOCATION (HUMAN AND MODEL ORGANISMS)

- neXtProt and UniProtKB: No consensus between orthologs: Two transmembrane domains in the human protein, one in *M. musculus,* none in *D.rerio* and *X.laevis*.
- DeepLoc: cytoplasmic.
- WoLF PSORT: cytoplasmic (human, *D.rerio* and *X.laevis)* or plasma membrane (*M.musculus)*

TMEM232 is probably cytoplasmic and its name should probably be revised.

INTERACTIONS (HUMAN)

NextProt: None of high quality

EXPRESSION (HUMAN AND MODEL ORGANISMS)

neXtProt: Validated at protein level: 3 unique peptides longer than 9 aa detected by mass spectrometry in different samples

LITERATURE

In *D. rerio*, induced by foxj1, a winged-helix transcription factor that regulates motile cilia biogenesis (1).

HPA

No high quality data in human at the protein level
RNAseq: enhanced in fallopian tube, pituitary gland and testis

GENEVESTIGATOR

- **Human microarrays**: medium to high in respiratory epithelium. Medium in male and female reproductive organs, nervous system structures and cells, bone marrow endothelium, progenitor cell, renal tubulointerstitium and epidermal melanocyte.
- **Human RNAseq**: high in respiratory epithelium, brain structures and cells, testis, fallopian tube, colonic microvessel endothelium and synovial membrane macrophage. Medium in all systems.
- **Mouse microarrays**: High in sperm cells, testis, oocyte and trachea. Medium in most of the systems.
- RNAseq: High in testis, brain structures and cells, as well as lung.

COEXPRESSION (HUMAN)

GENEVESTIGATOR

cilium assembly and organization, cilium movement, and determination of left/right symmetry

SEEK

cilium movement, cilium assembly and organization and protein localization to cilium

PHENOTYPES/DISEASES (HUMAN AND ORTHOLOGS)

- rs17513503 (upstream of TMEM232 and downstream of the slightly overlapping gene SLC25A46): allergic rhinitis and grass sensitization (2).
- rs11357450 (intronic deletion) and rs7701890 (intronic SNP): susceptibility to atopic dermatitis in the Chinese Han

population (3-4).

- rs9326801 (intronic SNP) : susceptibility to atopic dermatitis in the Japanese population (5).

PREDICTED FUNCTIONS

The results of our expression and coexpression analyses, the induced expression by foxj1 in Zebrafish and the allergic rhinitis association suggest that TMEM232 may have a role in **motile cilia assembly or function**. Indeed, impaired mucociliary clearance has been described in chronic airway inflammatory diseases (6) and ciliary abnormalities have been described in patients with allergic and chronic rhinitis (7-8). The associations with atopic dermatitis may indicate an additional role of TMEM232 in **non-motile cilia assembly**, as those cilia participate in epidermal stress responses, homeostasis of the interfollicular epidermis and normal keratinocyte differentiation (9). TMEM232 may also be involved in **protein localization to cilium**, which may be important for cilium assembly, cilium movement, or signaling events occurring at the cilium.

ADDITIONAL COMMENTS

Wrongly described in the literature as a tetraspan transmembrane protein (10).

REFERENCES

1. Choksi, S. P., Babu, D., Lau, D., et al. (2014) Systematic discovery of novel ciliary genes through functional genomics in the zebrafish. *Dev.*, **141**, 3410–3419.
2. Ramasamy, A., Curjuric, I., Coin, L. J., et al. (2011) A genome-wide meta-analysis of genetic variants associated with allergic rhinitis and grass sensitization and their interaction with birth order. *J. Allergy Clin. Immunol.*, **128**, 996–1005.
3. Wu, Y. Y., Tang, J. P., Liu, Q., et al. (2017) Scanning indels in the 5q22.1 region and identification of the TMEM232 susceptibility gene that is associated with atopic dermatitis in the Chinese Han population. *Gene*, **617**, 17–23.
4. Shen, C., Liu, L., Jiang, Z., et al. (2015) Four genetic variants interact to confer susceptibility to atopic dermatitis in Chinese Han population. *Mol. Genet. Genomics*, **290**, 1493–1498.
5. Hirota, T., Takahashi, A., Kubo, M., et al. (2012) Genome-wide association study identifies eight new susceptibility loci for atopic dermatitis in the Japanese population. *Nat. Genet.*, **44**, 1222–1226.
6. Guan, W. jie, Peng, Y., Zi, X. xue, et al. (2018) Motile Ciliary Disorders in Chronic Airway Inflammatory Diseases: Critical Target for Interventions. Motile Ciliary Disorders in Chronic Airway Inflammatory Diseases: Critical Target for Interventions. *Curr. Allergy Asthma Rep.* (2018) , **18**.
7. Maurizi, M., Paludetti, G., Todisco, T., et al. (1984) Ciliary ultrastructure and nasal mucociliary clearance in chronic and allergic rhinitis. *Rhinology*, **22**, 233–240.
8. Peng, Y., Guan, W. J., Zhu, Z. C., et al. (2020) Microarray Assay Reveals Ciliary Abnormalities of the Allergic Nasal Mucosa. *Am. J. Rhinol. Allergy*, **34**, 50–58.
9. Croyle, M. J., Lehman, J. M., Connor, A. K. O., et al. (2011) Role of epidermal primary cilia in the homeostasis of skin and hair follicles. *Development*, **138**, 1675–1685.
10. Sun, L. D., Xiao, F. L., Li, Y., et al. (2011) Genome-wide association study identifies two new susceptibility loci for atopic dermatitis in the Chinese Han population. *Nature Genetics*, Vol. 43, pp. 690–694.
