# Supplementary material for: Functionathon: a manual data mining workflow to generate functional hypotheses for uncharacterized human proteins and its application by undergraduate students
Source: Database (Oxford). 2021 Jul 28;2021:baab046. doi: 10.1093/database/baab046 (PMC8317215; doi:10.1093/database/baab046)
Supplement: baab046_Supp [file baab046_supp.zip › Supp_File_S2.pdf]

Supplementary file S2

***Figure 1*.** Multiple sequence alignment of C6orf118 and orthologs. Conservation is indicated under the alignment. The Translin-associated factor X-interacting protein 1, N-terminal (IPR032755) domain is indicated with a red square, the coiled coil domains with green squares. The UniProt accession numbers of the sequences are as follows: *Alligator sinensis* A0A1U7RSV3*, Anas platyrhynchos* U3IR65*, Anolis carolinensis* H9G790*, Branchiostoma floridae* C3ZMD8*, Capitella teleta* R7VLG1*, Ciona intestinalis* F6QS01*, Homo sapiens* Q5T5N4*, Lottia gigantea* V4AD75*, Mus musculus* Q3TTN6*, Pelodiscus sinensis* K7FK90*, Salmo salar* A0A1S3NDW9*, Strongylocentrotus purpuratus* W4YG44*, Xenopus laevis* A0A1L8G278*, Xenopus tropicalis* Q0V9C4.

Figure 2. Multiple sequence alignment of C7orf25 and orthologs. Conservation is indicated under the alignment. Sequences are coloured by percentage of identity according to Jalview. Domains DUF5614 and DUF1308 are highlighted in green and red respectively. The positions of potentially phosphorylated residues in human C7orf25 are indicated by green circles. Position of the potential acidic catalytic residues are marked by red circles. Sequence accession numbers at UniProtB are the following: *Homo sapiens* Q9BPX7, *Mus Musculus* Q91WD4, Gallus gallus A0A1D5P7R3, *Danio rerio* Q803H0, *Xenopus tropicalis* Q5BKL1, Xenopus laevis Q08AW5, *Drosophila melanogaster* Q9W0M6, *Caenorabditis elegans* Q19987, *Arcella intermedia* A0A6B2L838, *Arabidopsis thaliana* A0A654EZB8, *Rhizopus microsporus* A0A1X0R3Z4, Trypanosome brucei Q387L1, *Tuber magnatum* A0A317T2G0, *Nostoc sp* K9QMI2, *Moumouvirus goulette* M1PC36.

Figure 3. **Sequence alignment analysis of CXorf58, MFI and orthologs. A**: Sequence alignment of human CXorf58 and human MFI proteins. Residues are colored according to Clustal default color scheme. Conservation is indicated under the alignment. **B**: Guide tree obtained from Clustal Omega alignment of CXorf58 and MFI orthologs **C:** Potential IQ domain magnification. Residues are colored according to Clustal default color scheme. **D:** Multiple sequence alignment of CXorf58 and MFI sequences. Conservation is indicated under the alignment. Sequences are coloured by percentage of identity according to Jalview. The position of the potential IQ domain is highlighted with a red box. UniProtKB accession numbers for CXorf58 are: *Homo sapiens* Q96LI9, *Mus musculus* A0A5F8MPE6, *Oncorhynchus_kisutch* A0A060WZF5, *Xenopus tropicalis* A8E5V6, *Octopus vulgaris* A0A0L8FJI4, *Amphimedon queenslandica* A0A1X7TW27, *Ciona intestinalis* F6PWJ2, *Stylophora pistillata* A0A2B4RI87, *Salpingoeca rosetta* F2U155, *Spizellomyces punctatus* A0A0L0HIZ9. UniProtKB or RefSeq

accession numbers for MFI are: *Homo sapiens:* Q8NCR3, *Amphimedon queenslandica:* A0A1X7VJ87, *Ciona intestinalis:* F6PP06, *Mytilus coruscus*: A0A0L8FYB0, *Xenopus tropicalis:* F7CTR5, *Danio rerio:* NP_001373428, *Paramecium tetraurelia:* A0CK10, *Tetrahymena thermophila:* XP_001030005, *Spizellomyces punctatus* A0A0L0HVN9.

Figure 4. **Multiple sequence alignment of RSRP1 and orthologs**. Conservation is indicated under the alignment. Sequences are coloured by percentage of identity according to Jalview. The phosphorylated Ser in human and the Ser in the orthologs in this region are indicated with red squares. The UniProtKB or RefSeq sequence accession numbers are as follows: *Alligator sinensis* A0A1U7RG00, *Danio rerio* Q1ECZ9, *Gallus gallus* A0A1D5PEB7, *Homo sapiens* Q9BUV0*, Mus musculus* Q3UC65, *Nanorana parkeri* XP_018413522.1, *Pelodiscus sinensis* K7FY35, *Rattus norvegicus* Q5U2S0, *Rhincodon typus* XM_020518674.1.

Figure 5. **Multiple sequence alignment and conservation analysis of SMLR1 and ADIG sequences. A:** Multiple sequence alignment of SMLR1 and orthologs. Conservation is indicated under the alignment. Sequences are coloured by percentage of identity according to Jalview. The transmembrane domains (for human those annotated in neXtProt, for the rest of the species predicted by TMHMM2.0) are indicated with red squares and with a yellow square the alternative initiation Met. **B:** Multiple sequence alignment of SMLR1 and ADIG. Conservation is indicated under the alignment. Sequences coloured by percentage of identity according to Jalview. Red rectangles indicate the transmembrane domain of the human proteins. **C:** Guide tree performed with Clustal Omega. UniProtKB or RefSeq accession numbers for ADIG sequences are the following: *Alligator mississippiensis* XP_019340173.1*, Anas platyrhynchos XP_012947641.2, Chelonoidis abingdonii XP_032630224.1, Fumlmarus glacialis* XP_009574066.1*, Homo sapiens* Q0VDE8*, Lacerta agilis* XP_033009272.1, *Macaca mulatta* F7B4E4*, Meleagris gallopavo* XP_019477718.1, *Mus musculus* Q8R400*, Rattus norvegicus* XP_032758330*.1.* UniProtKB or RefSeq accession numbers for SMLR1 sequences are the following *Alligator mississippiensis* A0A151M398, *Amblyraja radiata* XR_004412037.1, *Anas platyrhynchos* XP_021126027.1, *Anguilla anguilla* XP_035277921.1, *Chelonoidis abingdonii* XP_032628575.1, *Cyprinus carpio* KTG25365.1, *Danio rerio* XR_222668.4, *Gallus gallus* XR_211919.3, *Homo sapiens* H3BR10, *Lacerta agilis* XP_033001307.1, *Macaca mulatta* G7MQD8, *Mus musculus* J3QMJ4, *Nanorana parkeri NW_017308262.1, Nipponia nippon* XP_009459104.1, *Paramormyrops kingsleyae* XP_023652802.1, *Rattus norvegicus* M0R807, *Rhinatrema bivittatum* XP_029450422*, Salmo salar* NC_027305.1, *Xenopus tropicalis* XR_208637.4. SMLR1 protein sequences of *Amblyraja radiata, Nanorana parkeri, Salmo salar, Xenopus tropicalis*, *Danio rerio* and *Gallus gallus* are the translations obtained as the result of tBLASTn.

Figure 6. Multiple sequence alignment of TMEM53. Conservation is indicated under the alignment. Sequences coloured by percentage of identity according to Jalview. The putative catalytic sites based on our HHPRED and SWISS-MODEL analyses are highlighted with red squares. In green squares the predicted transmembrane domains according to TMHMM. The UniProtKB accession numbers are the following: *Anolis carolinensis* G1KQB8*, Arabidopsis thaliana* A0A178VZN4*, Branchiostoma floridae* C3ZJ88*, Caenorhabditis elegans(1)* Q17846*, Caenorhabditis elegans(2)* Q22750*, Caenorhabditis elegans(3)* Q9TZH8*, Capitella teleta* R7TA92*, Ciona savignyi* H2Z7C5*, Danio rerio* Q6DHN0*, Dictyostelium discoideum(1)* Q54YR8*, Dictyostelium discoideum(2)* Q54N85*, Drosophila melanogaster* A1Z6G9*, Gallus gallus* A0A1L1RPU6*, Homo sapiens* Q6P2H8*, Mus musculus* Q9D0Z3*, Nematostella vectensis* A7SHF2*, Octopus bimaculoides* A0A0L8GWT2*, Oryza sativa subsp. Japonica* Q6Z6I1*, Penicillium chrysogenum* B6HTA6*, Salpingoeca rosetta F2U052, Strongylocentrotus purpuratus(1)* W4YNQ6*, Strongylocentrotus purpuratus(2)* W4Y103*, Xenopus laevis(1)* Q5PPS7*, Xenopus laevis(2)* A0A1L8GM57*, Xenopus tropicalis* A9JSB6*.*

Figure 7: Multiple sequence alignment of TMEM232 and orthologs. Conservation is indicated under the alignment. Sequences were coloured by percentage of identity according to Jalview. The transmembrane domains predicted by Phobius for the human and mouse proteins are indicated with red rectangles. The UniProtKB accession numbers are the following: *Homo sapiens* C9JQI7, *Mus musculus* Q5K6N0, *Alligator mississippiensis* A0A151PIJ1, *Anolis carolinensis* H9GC94, *Xenopus laevis* A0A1L8I1Q0, *Danio rerio* A5WVS1, *Capitella teleta* R7TJS3, *Branchiostoma floridae* C3YDS1, *Lottia gigantea* V3ZU21, *Ciona intestinalis* F6SFR5, *Macrostomum lignano* A0A267E638, *Trichoplax adhaerens* B3RTI4, *Nematostella vectensis* A7S2I4, *Amphimedon queenslandica* A0A1X7U5R7, *Strongylocentrotus purpuratus* W4Y6N3.

Figure 1

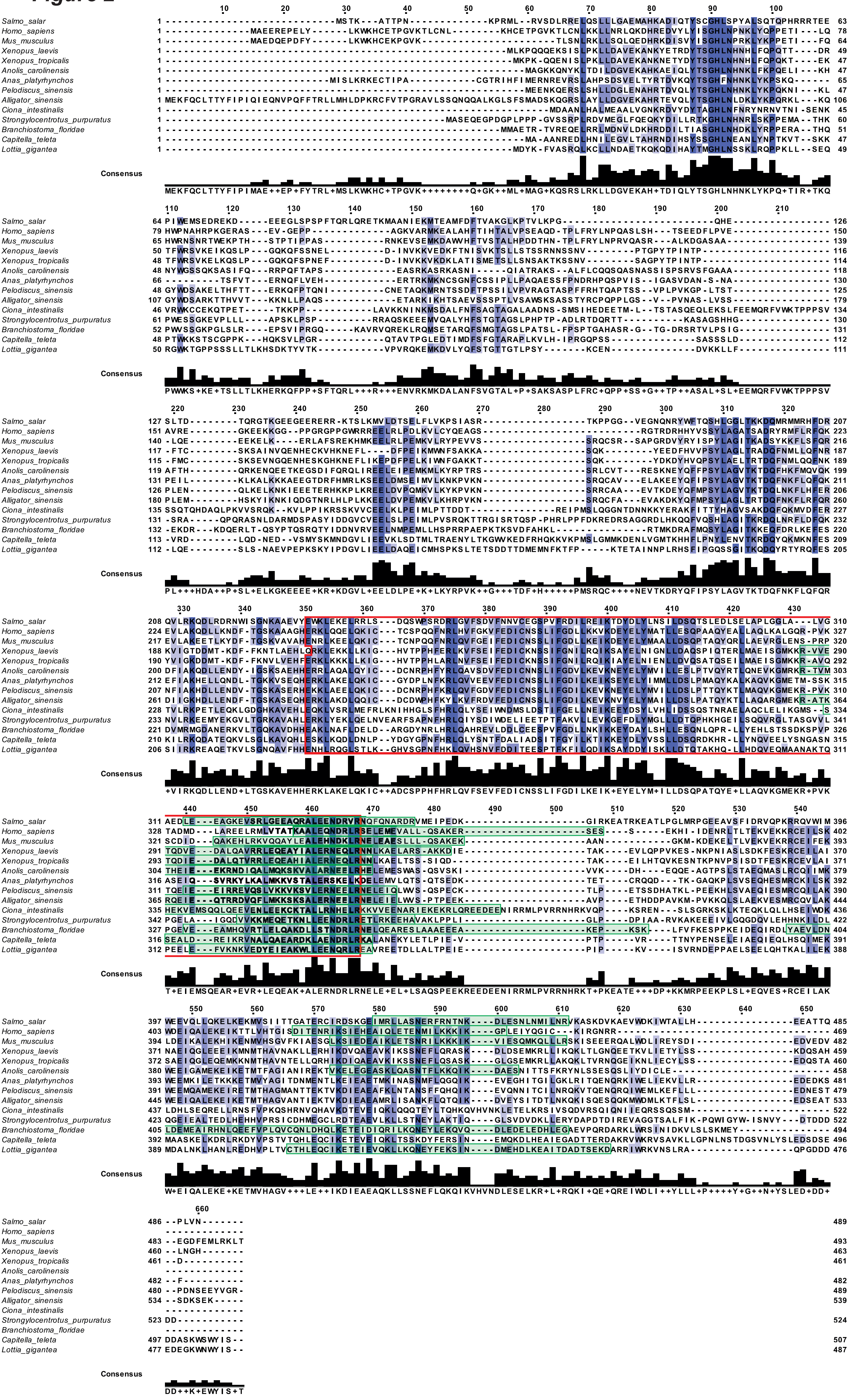
Salmo_salar
Homo_sapiens
Mus_musculus
Xenopus_laevis
Xenopus_tropicalis
Anolis_carolinensis
Anas_platyrhynchos
Pelodiscus_sinensis
Alligator_sinensis
Ciona_intestinalis
Strongylocentrotus_purpuratus
Branchiostoma_floridae
Capitella_teleta
Lottia_gigantea
Consensus

Figure 2

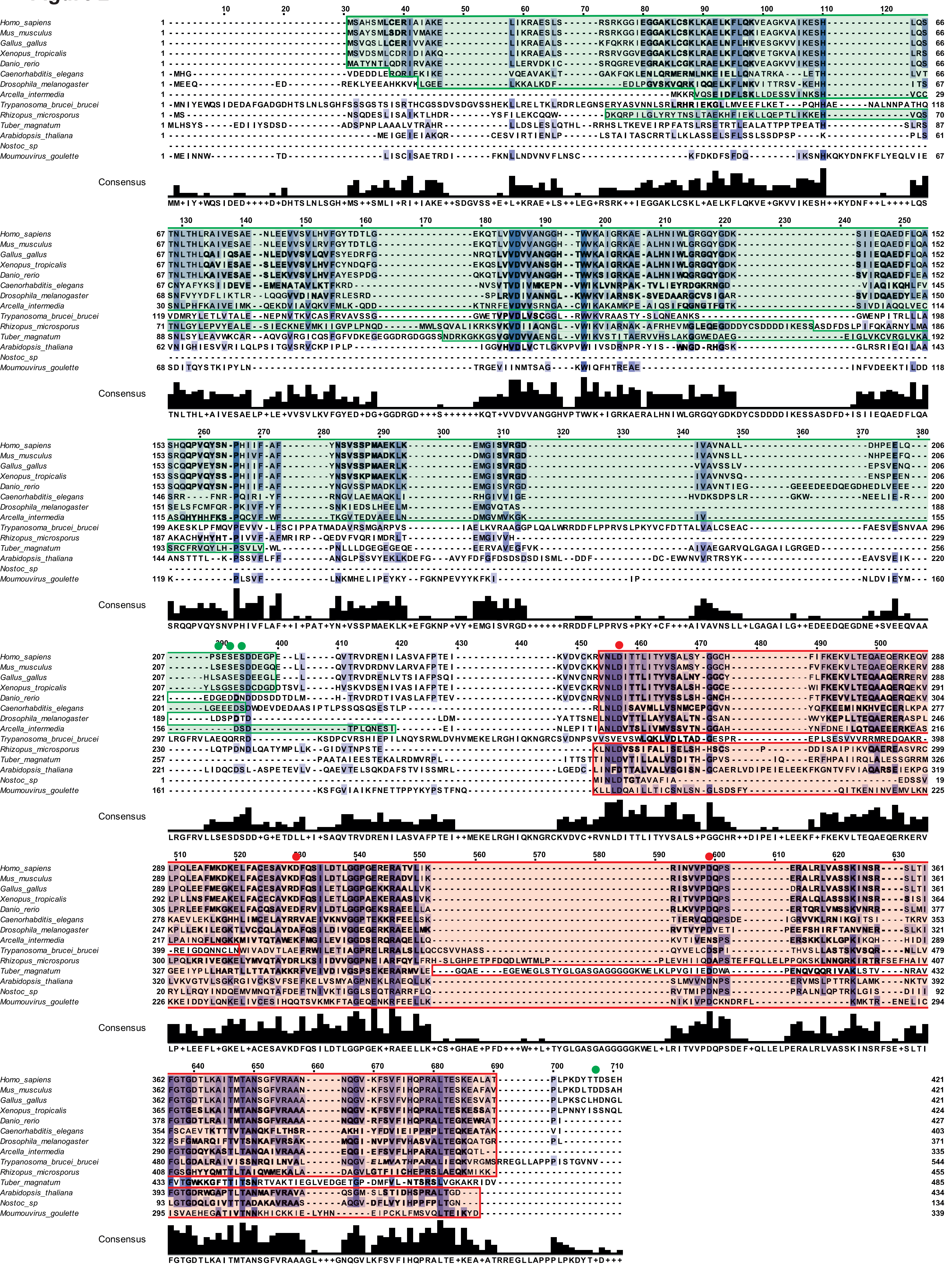

Homo_sapiens
Mus_musculus
Gallus_gallus
Xenopus_tropicalis
Danio_rerio
Caenorhabditis_elegans
Drosophila_melanogaster
Arcella_intermedia
Trypanosoma_brucei_brucei
Rhizopus_microsporus
Tuber_magnatum
Arabidopsis_thaliana
Nostoc_sp
Moumouvirus_goulette
Consensus

Figure 3

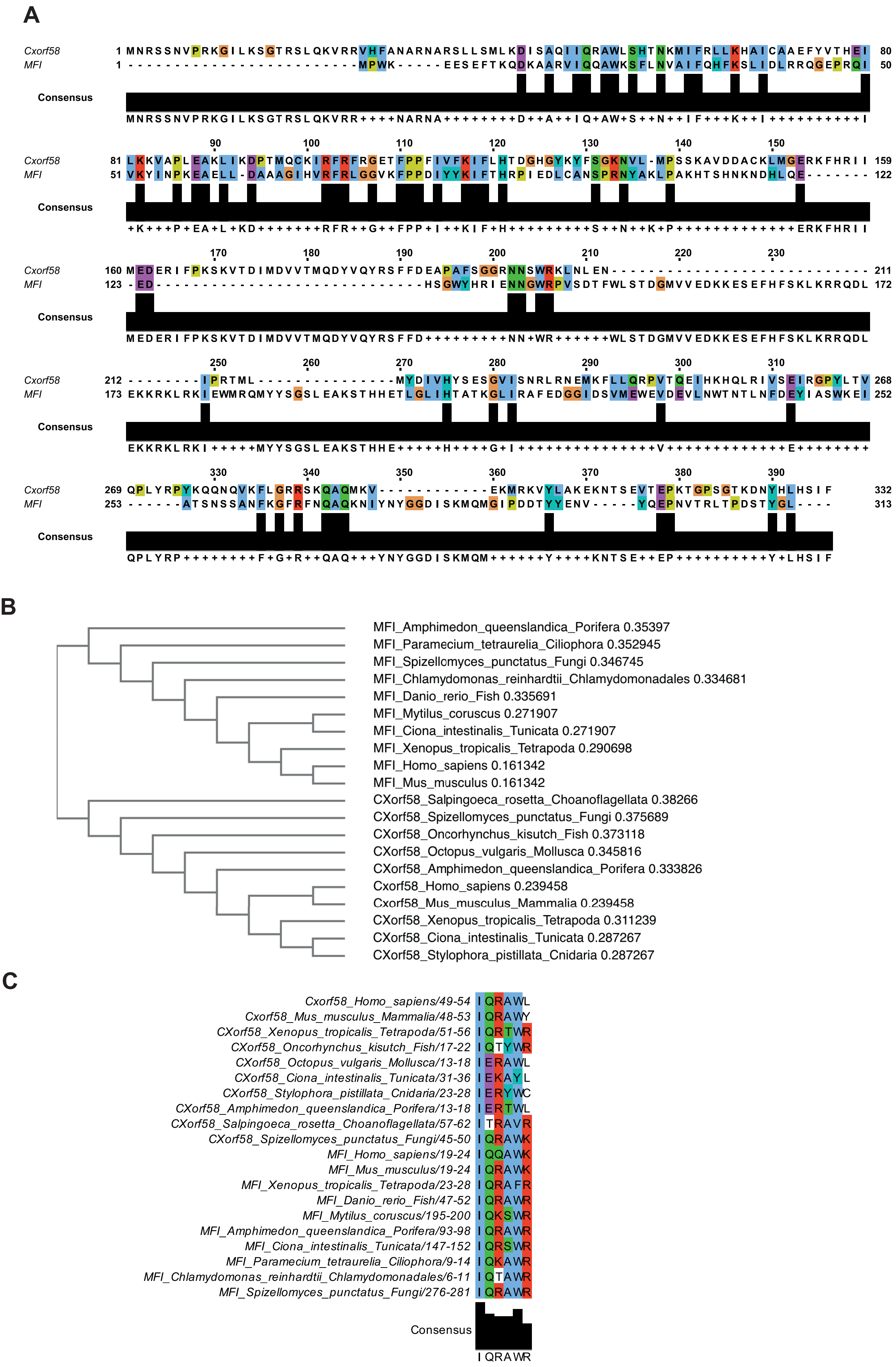
A
Cxorf58
MFI
Consensus
B
MFI_Amphimedon_queenslandica_Porifera 0.35397
MFI_Paramecium_tetraurelia_Ciliophora 0.352945
MFI_Spizellomyces_punctatus_Fungi 0.346745
MFI_Chlamydomonas_reinhardtii_Chlamydomonadales 0.334681
MFI_Danio_rerio_Fish 0.335691
MFI_Mytilus_coruscus 0.271907
MFI_Ciona_intestinalis_Tunicata 0.271907
MFI_Xenopus_tropicalis_Tetrapoda 0.290698
MFI_Homo_sapiens 0.161342
MFI_Mus_musculus 0.161342
CXorf58_Salpingoeca_rosetta_Choanoflagellata 0.38266
CXorf58_Spizellomyces_punctatus_Fungi 0.375689
CXorf58_Oncorhynchus_kisutch_Fish 0.373118
CXorf58_Octopus_vulgaris_Mollusca 0.345816
CXorf58_Amphimedon_queenslandica_Porifera 0.333826
Cxorf58_Homo_sapiens 0.239458
Cxorf58_Mus_musculus_Mammalia 0.239458
CXorf58_Xenopus_tropicalis_Tetrapoda 0.311239
CXorf58_Ciona_intestinalis_Tunicata 0.287267
CXorf58_Stylophora_pistillata_Cnidaria 0.287267
C
Cxorf58_Homo_sapiens/49-54 IQRAWL
Cxorf58_Mus_musculus_Mammalia/48-53 IQRAWY
CXorf58_Xenopus_tropicalis_Tetrapoda/51-56 IQRTWR
CXorf58_Oncorhynchus_kisutch_Fish/17-22 IQTYWR
CXorf58_Octopus_vulgaris_Mollusca/13-18 IERAWL
CXorf58_Ciona_intestinalis_Tunicata/31-36 IEKAYL
CXorf58_Stylophora_pistillata_Cnidaria/23-28 IERYWC
CXorf58_Amphimedon_queenslandica_Porifera/13-18 IERTWL
CXorf58_Salpingoeca_rosetta_Choanoflagellata/57-62 ITRAVR
CXorf58_Spizellomyces_punctatus_Fungi/45-50 IQRAWK
MFI_Homo_sapiens/19-24 IQQAWK
MFI_Mus_musculus/19-24 IQRAWK
MFI_Xenopus_tropicalis_Tetrapoda/23-28 IQRAFR
MFI_Danio_rerio_Fish/47-52 IQRAWR
MFI_Mytilus_coruscus/195-200 IQKSWR
MFI_Amphimedon_queenslandica_Porifera/93-98 IQRAWR
MFI_Ciona_intestinalis_Tunicata/147-152 IQRSWR
MFI_Paramecium_tetraurelia_Ciliophora/9-14 IQKAWR
MFI_Chlamydomonas_reinhardtii_Chlamydomonadales/6-11 IQTAWR
MFI_Spizellomyces_punctatus_Fungi/276-281 IQRAWR
Consensus
IQRAWR

Figure 3

D

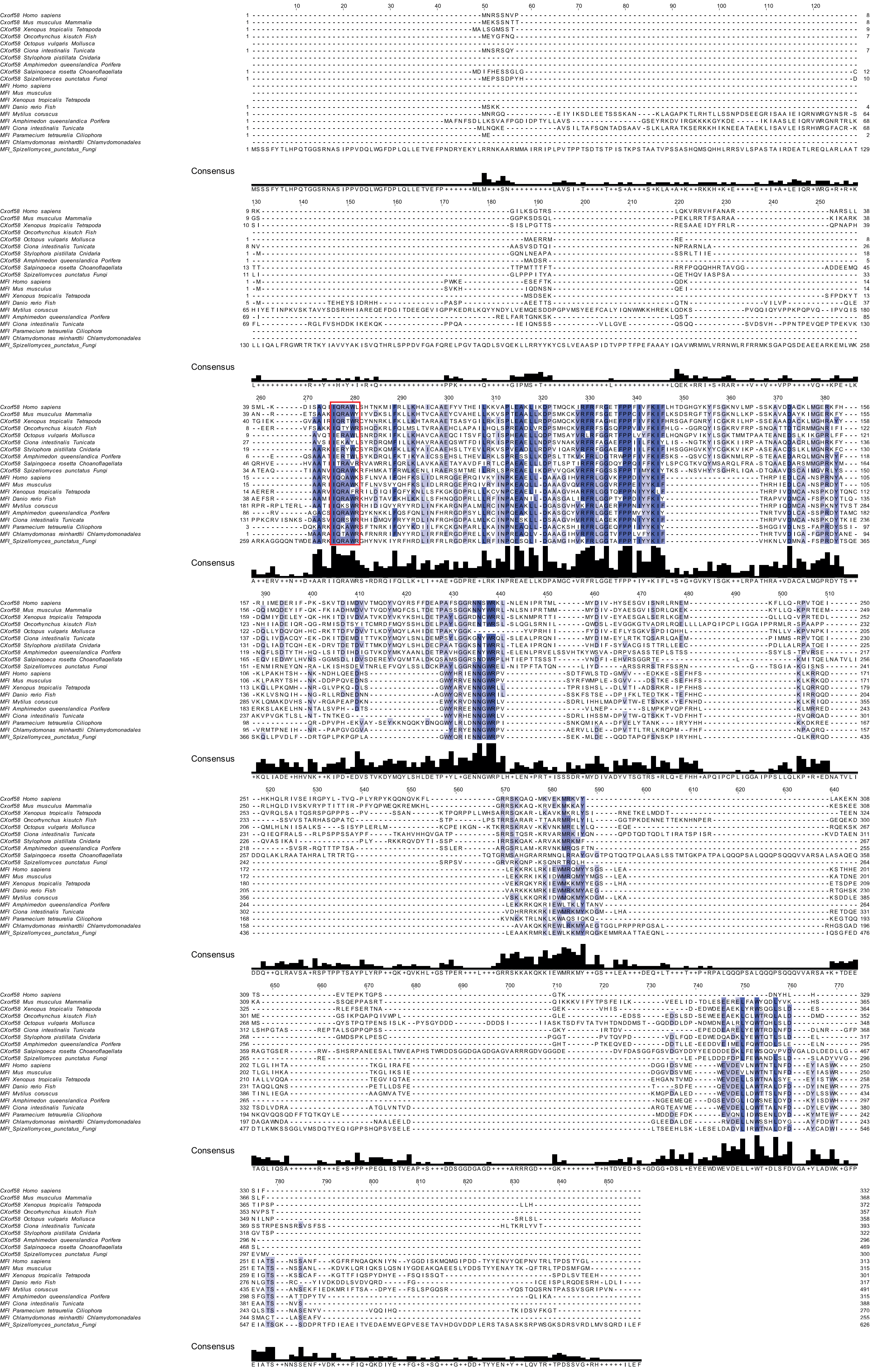

Figure 4

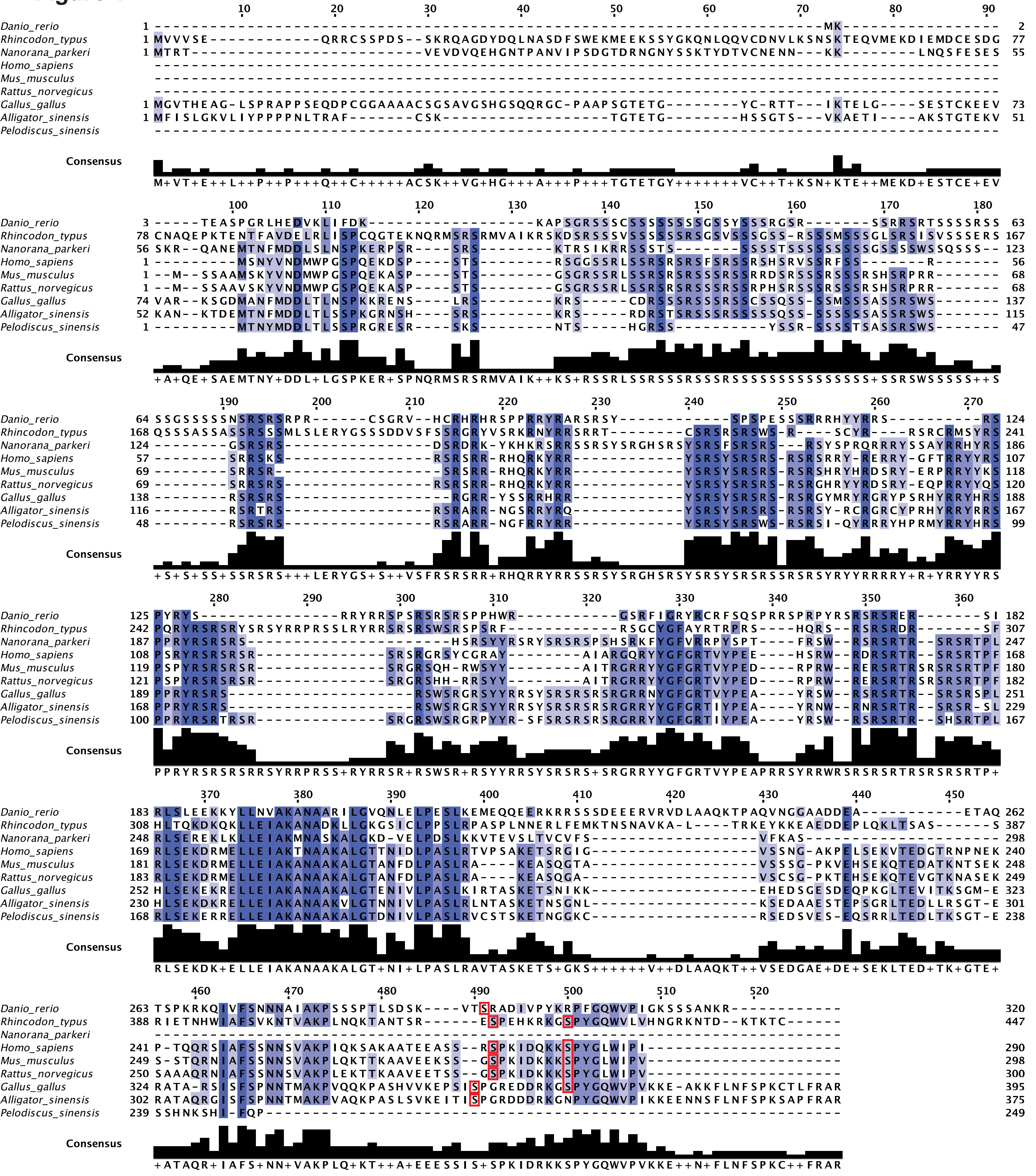

Figure 5

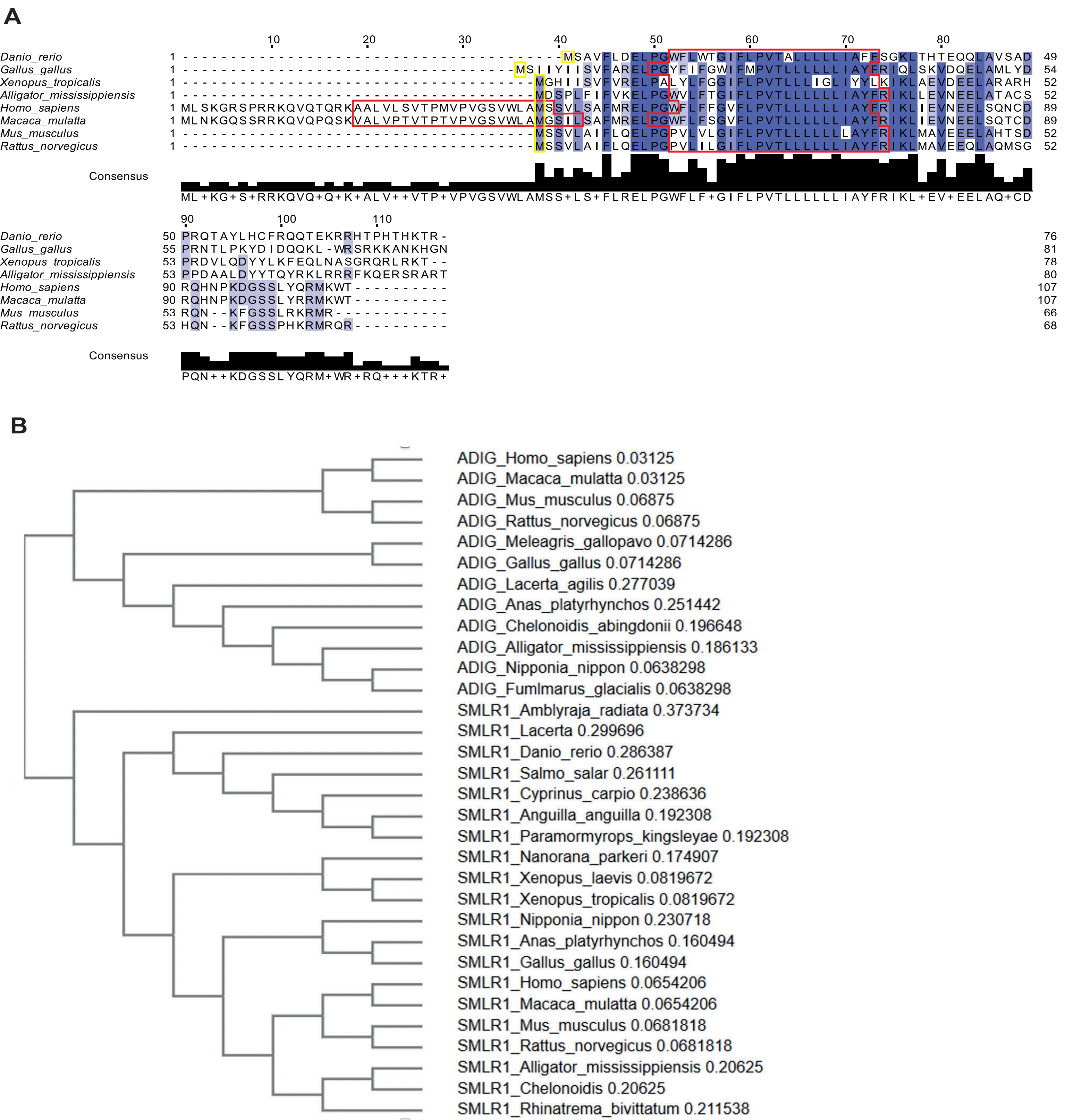
A
Danio_rerio
Gallus_gallus
Xenopus_tropicalis
Alligator_mississippiensis
Homo_sapiens
Macaca_mulatta
Mus_musculus
Rattus_norvegicus
Consensus
B
ADIG_Homo_sapiens 0.03125
ADIG_Macaca_mulatta 0.03125
ADIG_Mus_musculus 0.06875
ADIG_Rattus_norvegicus 0.06875
ADIG_Meleagris_gallopavo 0.0714286
ADIG_Gallus_gallus 0.0714286
ADIG_Lacerta_agilis 0.277039
ADIG_Anas_platyrhynchos 0.251442
ADIG_Chelonoidis_abingdonii 0.196648
ADIG_Alligator_mississippiensis 0.186133
ADIG_Nipponia_nippon 0.0638298
ADIG_Fumlmarus_glacialis 0.0638298
SMLR1_Amblyraja_radiata 0.373734
SMLR1_Lacerta 0.299696
SMLR1_Danio_rerio 0.286387
SMLR1_Salmo_salar 0.261111
SMLR1_Cyprinus_carpio 0.238636
SMLR1_Anguilla_anguilla 0.192308
SMLR1_Paramormyrops_kingsleyae 0.192308
SMLR1_Nanorana_parkeri 0.174907
SMLR1_Xenopus_laevis 0.0819672
SMLR1_Xenopus_tropicalis 0.0819672
SMLR1_Nipponia_nippon 0.230718
SMLR1_Anas_platyrhynchos 0.160494
SMLR1_Gallus_gallus 0.160494
SMLR1_Homo_sapiens 0.0654206
SMLR1_Macaca_mulatta 0.0654206
SMLR1_Mus_musculus 0.0681818
SMLR1_Rattus_norvegicus 0.0681818
SMLR1_Alligator_mississippiensis 0.20625
SMLR1_Chelonoidis 0.20625
SMLR1_Rhinatrema_bivittatum 0.211538

Figure 5

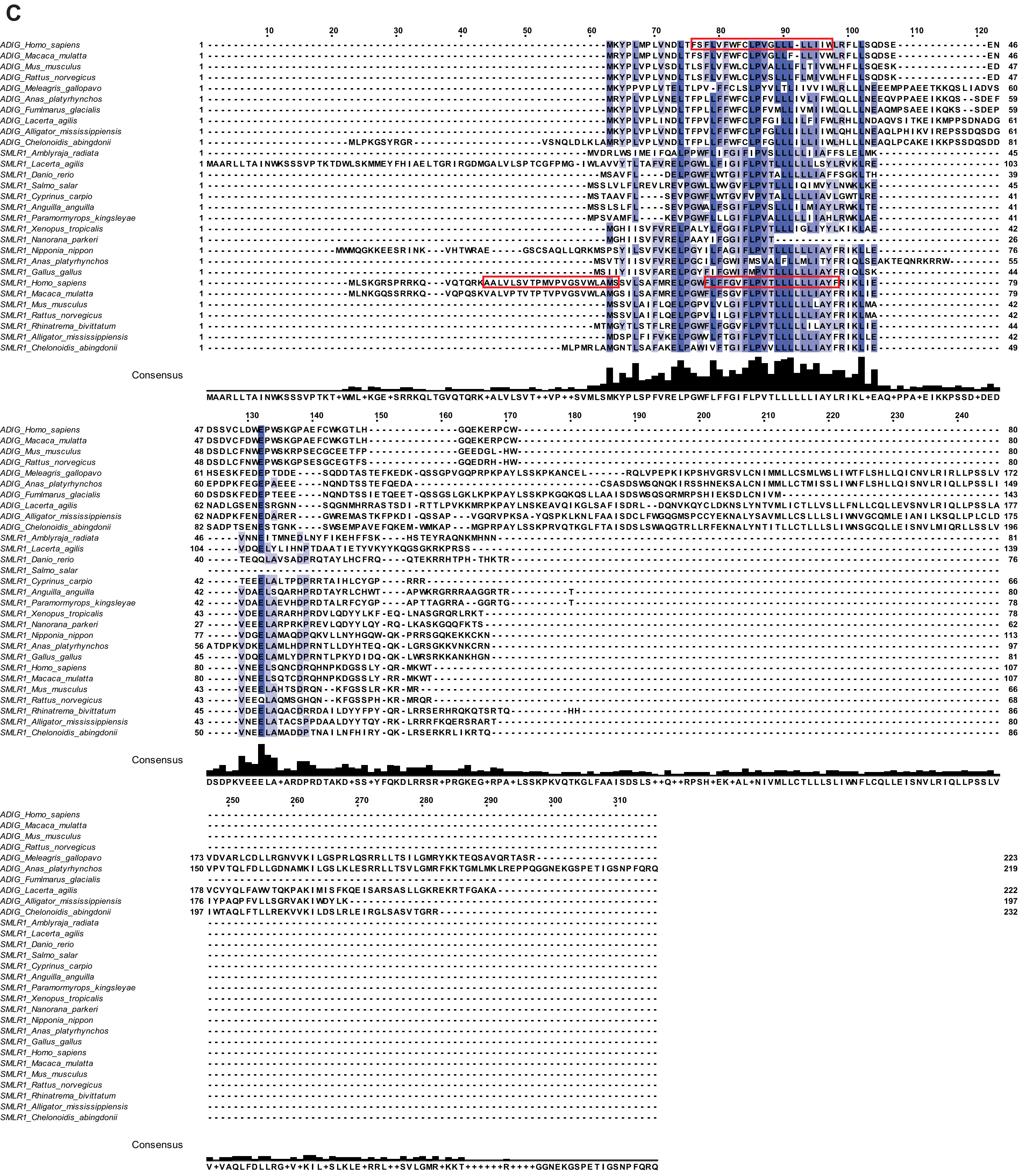
C
ADIG_Homo_sapiens
ADIG_Macaca_mulatta
ADIG_Mus_musculus
ADIG_Rattus_norvegicus
ADIG_Meleagris_gallopavo
ADIG_Anas_platyrhynchos
ADIG_Fumlmarus_glacialis
ADIG_Lacerta_agilis
ADIG_Alligator_mississippiensis
ADIG_Chelonoidis_abingdonii
SMLR1_Amblyraja_radiata
SMLR1_Lacerta_agilis
SMLR1_Danio_rerio
SMLR1_Salmo_salar
SMLR1_Cyprinus_carpio
SMLR1_Anguilla_anguilla
SMLR1_Paramormyrops_kingsleyae
SMLR1_Xenopus_tropicalis
SMLR1_Nanorana_parkeri
SMLR1_Nipponia_nippon
SMLR1_Anas_platyrhynchos
SMLR1_Gallus_gallus
SMLR1_Homo_sapiens
SMLR1_Macaca_mulatta
SMLR1_Mus_musculus
SMLR1_Rattus_norvegicus
SMLR1_Rhinatrema_bivittatum
SMLR1_Alligator_mississippiensis
SMLR1_Chelonoidis_abingdonii
Consensus

Figure 6

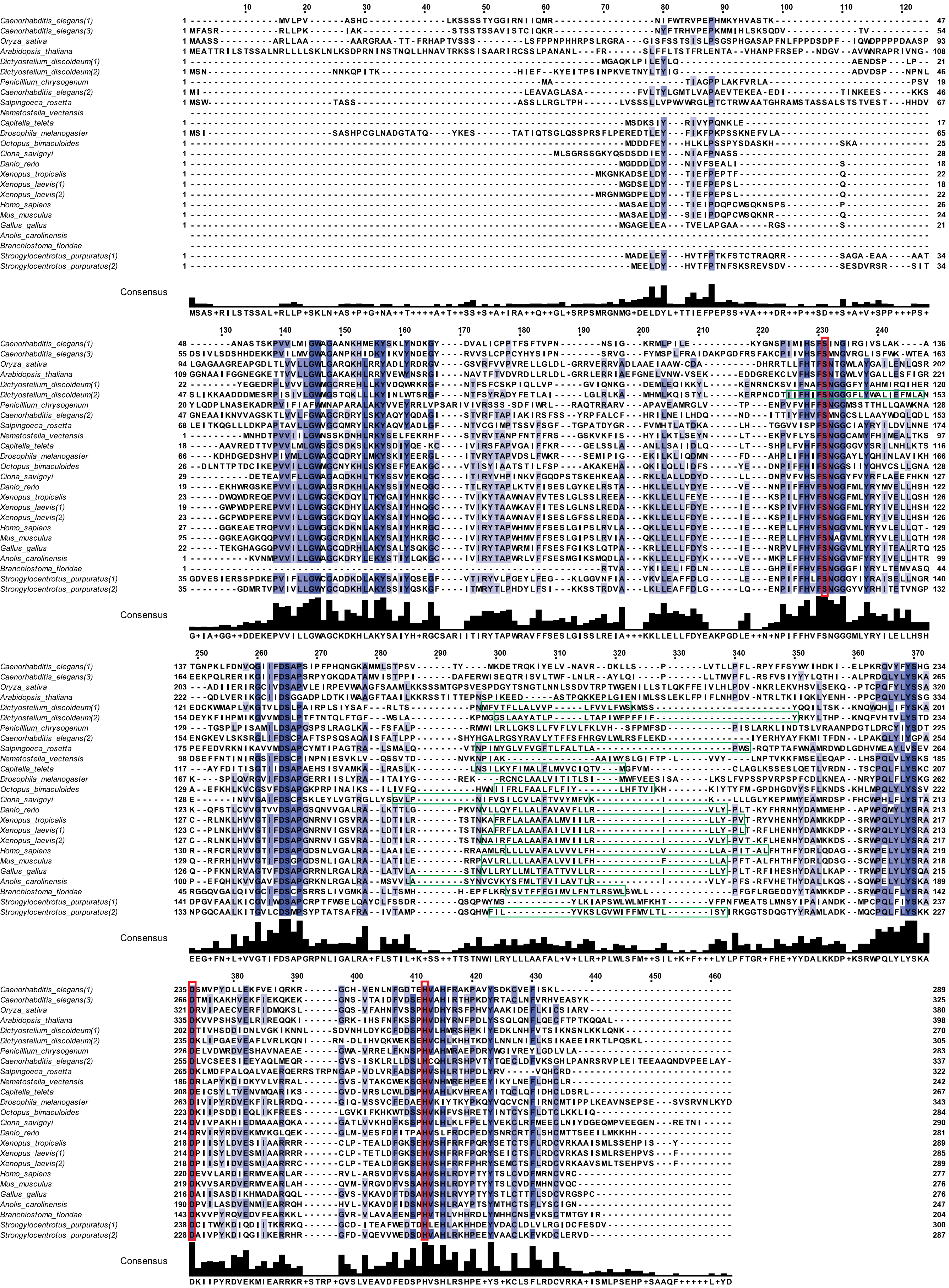
Caenorhabditis_elegans(1)
Caenorhabditis_elegans(3)
Oryza_sativa
Arabidopsis_thaliana
Dictyostelium_discoideum(1)
Dictyostelium_discoideum(2)
Penicillium_chrysogenum
Caenorhabditis_elegans(2)
Salpingoeca_rosetta
Nematostella_vectensis
Capitella_teleta
Drosophila_melanogaster
Octopus_bimaculoides
Ciona_savignyi
Danio_rerio
Xenopus_tropicalis
Xenopus_laevis(1)
Xenopus_laevis(2)
Homo_sapiens
Mus_musculus
Gallus_gallus
Anolis_carolinensis
Branchiostoma_floridae
Strongylocentrotus_purpuratus(1)
Strongylocentrotus_purpuratus(2)
Consensus

Figure 7

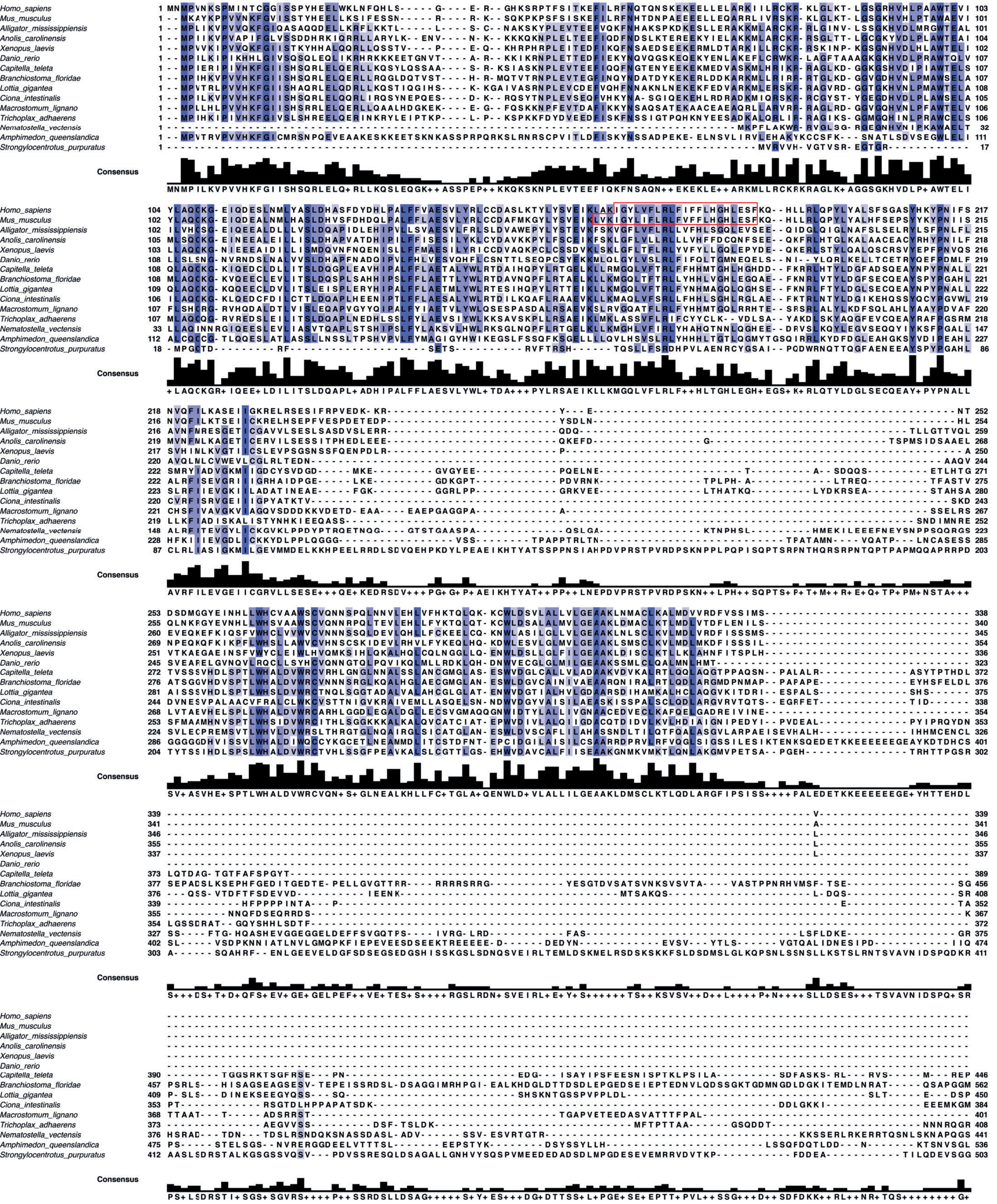
Homo_sapiens
Mus_musculus
Alligator_mississippiensis
Anolis_carolinensis
Xenopus_laevis
Danio_rerio
Capitella_teleta
Branchiostoma_floridae
Lottia_gigantea
Ciona_intestinalis
Macrostomum_lignano
Trichoplax_adhaerens
Nematostella_vectensis
Amphimedon_queenslandica
Strongylocentrotus_purpuratus
Consensus

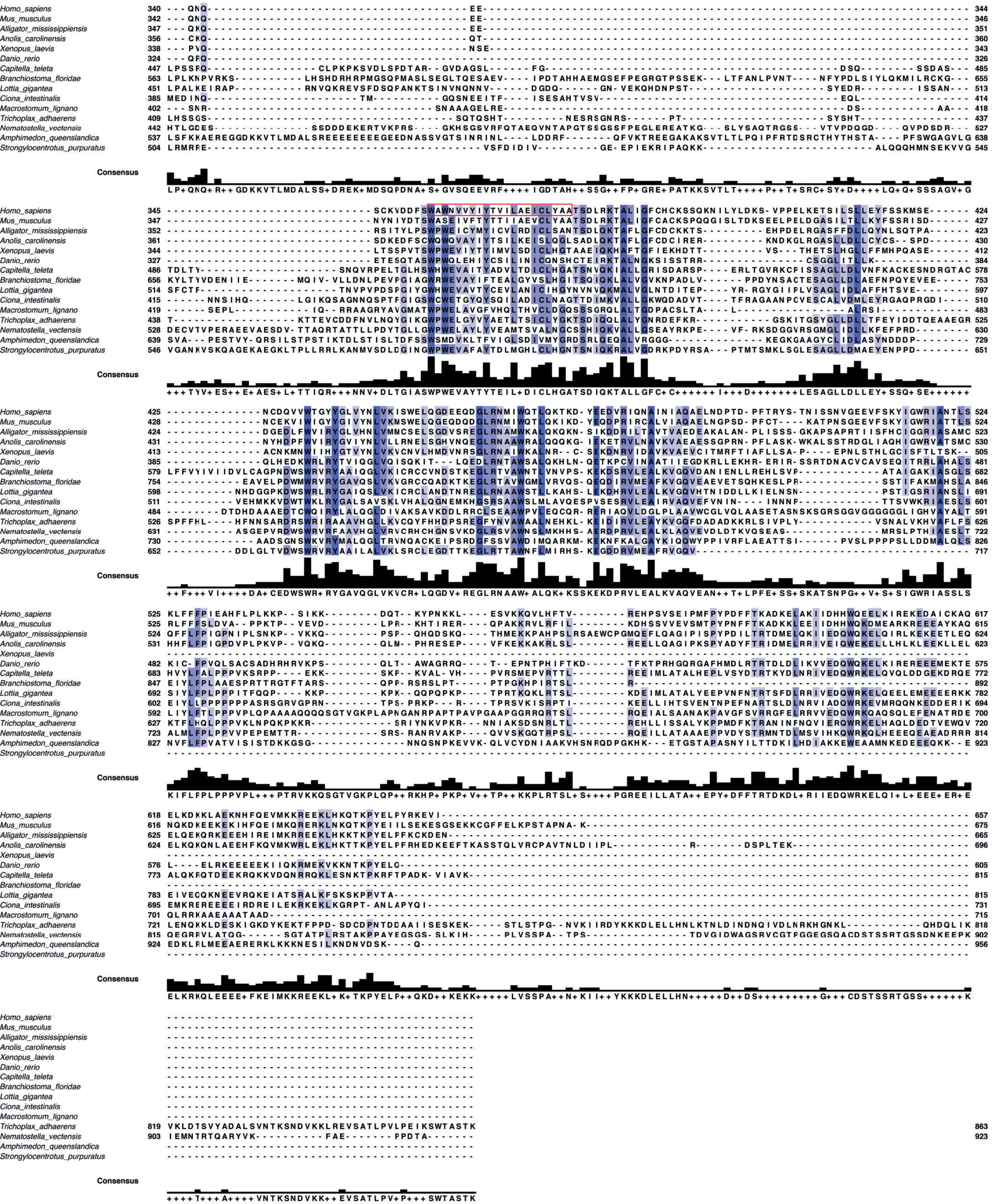

Homo_sapiens
Mus_musculus
Alligator_mississippiensis
Anolis_carolinensis
Xenopus_laevis
Danio_rerio
Capitella_teleta
Branchiostoma_floridae
Lottia_gigantea
Ciona_intestinalis
Macrostomum_lignano
Trichoplax_adhaerens
Nematostella_vectensis
Amphimedon_queenslandica
Strongylocentrotus_purpuratus
Consensus
